# Machine Learning-Based Estimation of Sports Performance in Elite Athletes

**DOI:** 10.3390/sports14100431

**Published:** 2026-10-04

**Authors:** Titanilla Takács, Gyula Bohus, Márton Tokodi, Emese Csulak, Attila Kovács, Ádám Szijártó, Kamilla Szabó, Luca Bátai, Zoltán Tősér, Hajnalka Vágó, Liliána Erzsébet Szabó, Dávid Becker, Béla Merkely, Nóra Sydó

**Affiliations:** 1 Heart and Vascular Center, Semmelweis University, 1122 Budapest, Hungary; takacstitanilla@gmail.com (T.T.); gyuszkob@gmail.com (G.B.); tokodi.marton@semmelweis.hu (M.T.); csulak.emese@gmail.com (E.C.); kovacs.attila3@semmelweis.hu (A.K.); szijarto.adam@semmelweis.hu (Á.S.); batai.luca.kata@semmelweis.hu (L.B.); vago.hajnalka@semmelweis.hu (H.V.); sz.liliana.e@gmail.com (L.E.S.); becker.david@semmelweis.hu (D.B.); merkely.bela@semmelweis.hu (B.M.); 2 Department of Cardiology, Petz Aladár University Teaching Hospital, 9024 Győr, Hungary; 3 Argus Cognitive, Inc., Lebanon, NH 03766-1441, USA; zoltan@arguscognitive.com; 4 Department of Sports Medicine, Semmelweis University, 1122 Budapest, Hungary

**Keywords:** sports cardiology, machine learning, sports performance, achievement, endurance

## Abstract

Achievements and endurance represent distinct aspects of sports performance, influenced by different underlying factors. However, these determinants are not well characterized. To address this gap between achievements and endurance, we evaluated machine learning models to estimate the competition results-based Achievement Score and the absolute cardiorespiratory capacity-based Endurance Score in elite athletes. We analyzed these models using SHapley Additive exPlanations to identify the most influential determinants of each performance component. Altogether, 688 healthy, asymptomatic elite athletes (19.7 ± 6.9 years; males: 75%) with 1194 sports cardiology screening exams were included in our machine learning-based analysis. In a hold-out test set of randomly selected 100 athletes (21.5 ± 9.2 years, males 67%), the best-performing models predicted Achievement Score and Endurance Score with the coefficients of determination values of 0.29 (95% bootstrap CI (0.00, 0.48)) and 0.69 (95% bootstrap CI (0.56, 0.79)), respectively. For Achievement Score, the strongest determinants were training years, weekly training hours, and age. In contrast, Endurance Score was primarily driven by skeletal muscle mass, weight, and peak heart rate. These findings suggest that achievements are predominantly related to training history and experience, whereas endurance is more closely associated with body composition and sports adaptation, partly reflecting its reliance on absolute cardiorespiratory measures.

## 1. Introduction

The main indicators of sports performance are achievements, which is represented by the medals lying on scores, times, or rankings. However, medals do not always reflect the best endurance, as in certain sports, technical, tactical skills, and teamwork are also important. Therefore, to describe sports performance, achievements and endurance have to be assessed separately. As excellent cardiorespiratory fitness is essential for all sports, the utilization of mental and physical abilities under fatigue can only be achieved with great endurance [1].

Outstanding endurance requires specific adaptation of the cardiopulmonary and muscular systems [2]. This adaptation can be assessed during sports cardiology screening. Numerous variables have been identified as contributing to endurance performance, including lactate threshold, glycolysis capacity, fat metabolism, mitochondrial biogenesis, hydration status, and recovery speed. Nevertheless, the most widely used parameter to assess cardiorespiratory fitness is maximum oxygen uptake (VO_2max_) [3,4]. The VO_2max_ is defined as the maximum rate of oxygen consumption that the body can absorb and utilize during intensive exercise [4]. Numerous studies have been conducted to estimate VO_2max_; however, cardiopulmonary exercise testing (CPX) remains the most precise method for evaluating cardiorespiratory capacity [5].

While VO_2max_ is considered the most significant determinant of endurance, limited data exist on the relative importance of other parameters that may also influence cardiorespiratory fitness. The analysis of these parameters can be challenging due to the interplay of numerous factors; however, artificial intelligence (AI) can provide valuable support for complex decision-making [6]. The field of AI is advancing rapidly, with its applications being increasingly explored in various areas of sports, including calculating expected goals in soccer, analyzing ball possession phases, analyzing training load before game day in basketball, and identifying tactical patterns [6,7]. Ensemble methods such as random forests, gradient boosting and support vector regressors are increasingly applied to multivariate physiological data in endurance sport [8]. Categorical Boosting and eXtreme Gradient Boosting (XGBoost) models trained on CPX, and anthropometry have predicted seasonal reinjury in elite soccer players more accurately than conventional statistics [9]. A genetic algorithm–optimized XGBoost classifier has assigned graded cardiopulmonary-endurance classes from cardiovascular, ventilatory and metabolic CPX indices [10]. Combined with Shapley Additive exPlanations (SHAP), Light Gradient-Boosting Machine and XGBoost have identified high-performing team-sport athletes from a diverse set of variables with VO_2max_ as the leading feature [11,12]. The determining factors of excellent cardiorespiratory fitness and endurance have been studied extensively; however, there is no clear evidence of how these physiological determinants contribute to elite-level athletic performance and research on the differences between athletic achievements and endurance remains limited.

Our study aimed to identify the most important determinants of sports performance assessed during comprehensive sports cardiology screening. Using AI-based analyses, we investigated and compared the key factors associated with athletic achievements and endurance. We hypothesized that achievements and endurance should be evaluated separately to provide a more comprehensive understanding of sports performance. This approach may help athletes monitor their preparation more effectively and optimize training efficiency.

## 2. Materials and Methods

### 2.1. Sports Cardiology Screening

The sports cardiology screening consisted of the following examinations: a sports-specific personal and family history questionnaire, including data on the best-achieved results at the time of the screening; detailed laboratory tests; a resting electrocardiogram (ECG); transthoracic echocardiography; body-composition analysis; and treadmill CPX. The exams were similar to our previous study [13].

Our sport-specific questionnaire was designed to assess the athlete’s personal and family medical history, sports category, training history (years), number of training sessions (hours/week), and best-achieved sports successes. All athletes with known cardiovascular diseases or symptoms were excluded from the analyses.

In all athletes, a standard resting 12-lead ECG was recorded (CardioSoft PC, General Electric Healthcare, Helsinki, Finland) and analyzed according to the latest recommendations for athletes [14]. Altogether, 7 parameters were collected from the resting ECG, including resting heart rate (HR), the presence of any arrhythmia, conduction and repolarization abnormalities, left ventricular hypertrophy signs, PQ segment length, and QRS width. Resting blood pressure was also measured.

Detailed laboratory tests were performed for all athletes, analyzing 63 parameters, including qualitative and quantitative blood counts, renal and liver function, lipids, C-reactive protein, electrolyte levels, creatine kinase, an iron panel (ferritin, transferrin, transferrin saturation, soluble transferrin receptor), and vitamin D levels altogether.

The athlete’s weight and height were measured with an automatic stadiometer (InBody BSM370, InBody Co., Ltd., Seoul, Korea). Body-composition analysis was performed with InBody 770 by bioelectric impedance analysis (InBody Co., Ltd., Seoul, Korea) and measured the athletes’ skeletal muscle mass (SMM, kg), body fat mass (kg), percentage body fat (PBF, %), fat-free mass (kg; %), soft lean mass (kg), extracellular water (L) and body-mass index (kg/m^2^). A total of 27 parameters were collected, including segmental analysis.

Transthoracic echocardiographic examinations were performed to identify structural or functional cardiac disease by measuring ventricular, atrial, and aortic diameters and assessing ventricular and valvular function according to current guidelines [15] (GE Vingmed Ultrasound, E95 Ultrasound system, 4Vc-D probe, Horten, Norway). In our database we collected the following parameters: ejection fraction (%), left ventricle posterior wall and interventricular septum wall thickness (mm), right and left atrium diameter (mm), left ventricular end-systolic and end-diastolic diameters (mm), diameter of the aortic root and ascending aorta (mm), peak aortic valve velocity (cm/s), tricuspid annular plane systolic excursion (TAPSE, mm), and diastolic filling pattern (E/A).

To define cardiorespiratory fitness, we used a vita maxima incremental test on a treadmill ergometer (General Electric T-2100, Healthcare, Helsinki, Finland). Continuous ECG monitoring was used throughout the entire test. To analyze gas exchange, a breath-by-breath automatic cardiopulmonary exercise system (Respiratory Ergostik, Geratherm, Bad Kissingen, Germany) was used. Different sport-specific protocols were applied for athletes participating in aquatic sports or dryland sports. The examination began with a 1 min preparation phase, during which blood lactate (EKF Diagnostics, Wales, UK) was measured. The warm-up phase began with walking at 6 km/h for 2 min in aquatic sports and 1 min in dryland sports. It was followed by running at a constant speed (aquatic sports: 8 km/h, dryland sports: 10 km/h) with an increasing increment rate (aquatic sports: 1.5% every minute, dryland sports: 1% every minute). After the athletes reached maximal effort, the test continued with an active recovery phase consisting of walking for 1 min, followed by a passive recovery phase of sitting for 4 min. Fingertip lactate measurements (mmol/L) were performed every 2 min during the load phase, at peak exercise, and at the end of the recovery phase. Athletes were asked not to grab the handrail during the test. The anaerobic threshold was set manually by a physician using the Wasserman curves. The following parameters were evaluated: treadmill time (minute), resting HR (bpm), max HR, HR recovery (max HR-1 min HR after maximum load), HR reserve (max HR-resting HR), maximal oxygen uptake (VO_2max_; L/min; mL/kg/min), anaerobic threshold HR (bpm; %), anaerobic threshold VO_2_ percentage (%), respiratory rate (1/min), oxygen saturation (%), oxygen pulse (oxygen uptake per heartbeats; L/min), maximum ventilation (VE, L/min), tidal volume (VT, L), the ratio of minute ventilation to carbon dioxide production (VE/VCO_2_), the perceived exertion (Borg Scale 6–20) and the respiratory exchange ratio (RER). Tests were considered maximal if all the following requirements were met: a respiratory exchange ratio (the ratio between the produced carbon dioxide and oxygen) ≥ 1.1, the plateau phase occurred on the VO_2_ curve, and a predicted max HR% (maximum HR compared to the predicted age-related maximum HR) greater than 90. If a test was not considered a maximal test, it was excluded from the analysis.

### 2.2. Achievement Score

We created the Achievement Score (1–5) to categorize the level of success and compare athletes based on their best competition results. The Score was given based on the result obtained closest in time to our examination. Five points were given to those who were ranked from 1st to 8th place at the Olympic Games; four points for the European or World Championships’ top 8 athletes, three points to athletes ranked from 1 to 8 at national championships, two points for regional championship medalists, and one point to athletes without any achievement. The Score was validated by using the World Aquatics Points Scoring, which compares the swim time to base time to allow the comparison of the athletes’ results [16,17].

### 2.3. Endurance Score

**Figure 1:**
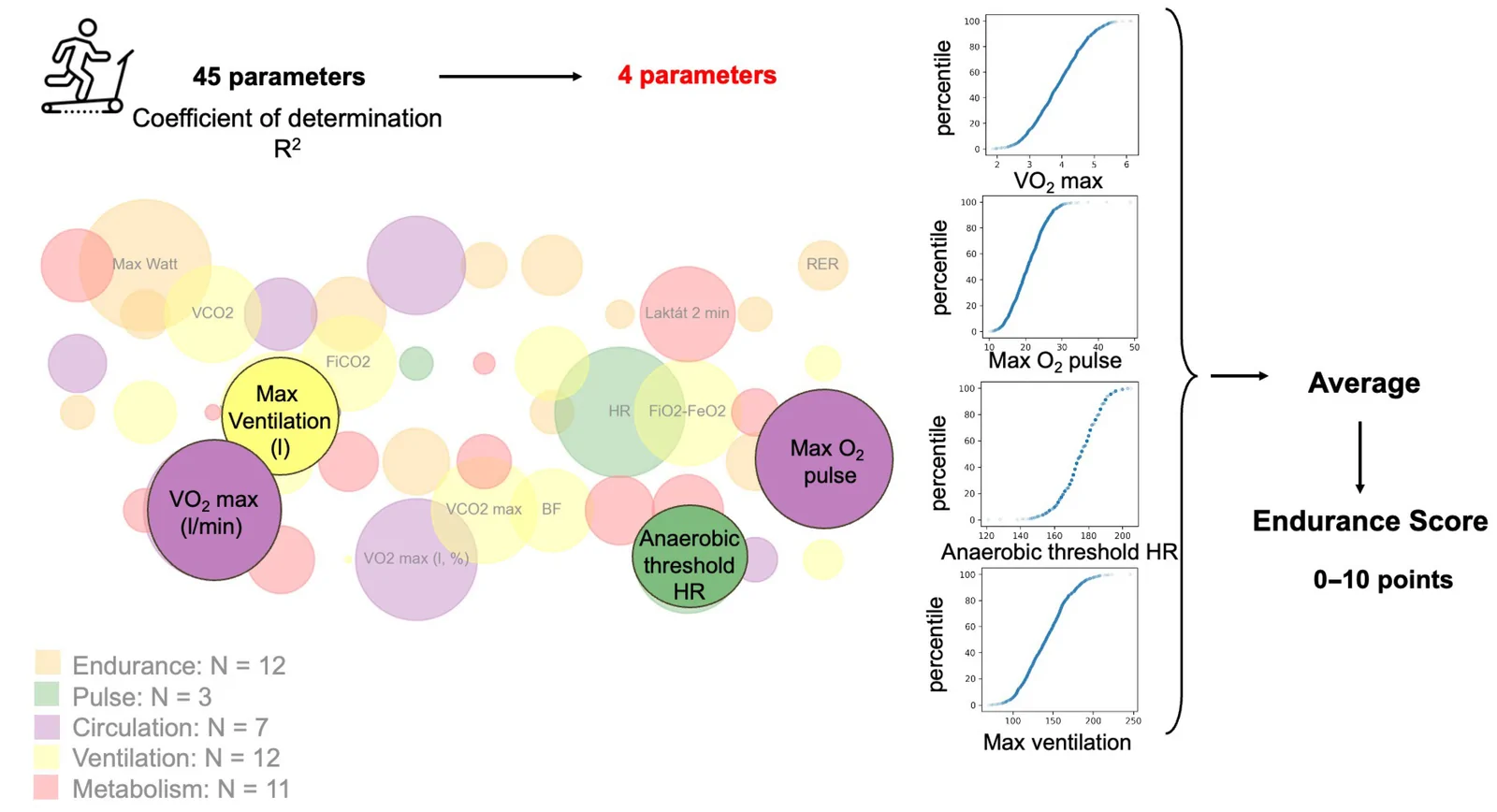
The establishment of the Endurance Score. The Endurance Score was defined based on 45 CPX parameters. The connection between these parameters was described by using the R^2^ values, which defines the strength of connection between them. We selected those parameters that can be estimated the most from non-CPX variables. The four most important determinants were chosen, ranked and by counting the average of percentile values we could create the Endurance Score. Abbreviations: O_2_: oxygen, HR: heart rate, VO_2max_: maximum oxygen consumption, R^2^: coefficient of determination.

We developed the Endurance Score that summarizes the results of the CPX examinations into a single parameter. Therefore, the Endurance Score represents and ranks athletes’ absolute cardiorespiratory capacity. In our database, we collected all the important parameters of CPX. Firstly, based on our experience and the relevant literature, we selected all potential parameters for the Score. Then, using the train cohort, we attempted to estimate them separately from the non-CPX variables (including patient history, resting ECG, echocardiography, and body-composition analysis parameters). We used the coefficient of determination (R^2^) to describe the strength of the connection between these CPX variables and non-CPX parameters. First, we selected variables based on relevant literature and expert opinion. We then excluded CPX parameters with low R^2^ scores and removed variables that relied on non-CPX parameters for calculation. Finally, 4 parameters-VO_2max_ (L/min), maximal oxygen pulse, anaerobic threshold HR, and maximal ventilation were chosen to describe the Endurance Score. Based on these parameters, the athletes were ranked by calculating their percentile position, and 0–10 points were assigned to each parameter. The average points assigned to these four parameters were calculated, yielding the Endurance Score for all athletes (Figure 1).

### 2.4. Study Population and Selection Criteria

We retrospectively analyzed competitive athletes who underwent sports cardiology screening at our centre. Inclusion criteria were: (i) competitive athlete registered with a sports club, academy or national team; (ii) screening performed on the same equipment; (iii) complete body-composition analysis; (iv) a treadmill CPX meeting the maximal-effort criteria defined in Section 2.1; and (v) availability of all four CPX endpoints used for the Endurance Score (VO_2max_, maximal oxygen pulse, maximal ventilation and HR at the first ventilatory threshold). Exclusion criteria were known cardiovascular disease or cardiovascular symptoms.

### 2.5. Machine Learning Model Development and Validation

All machine learning (ML) analyses were executed in Python 3 using the scientific stack NumPy and pandas. Model development relied on scikit-learn, XGBoost, SHAP, and joblib for serialization. Throughout the workflow, every random process was seeded (RANDOM_STATE = 42), all experiments were version-controlled, and progress was logged to support reproducibility.

Raw cardiopulmonary datasets were pre-filtered to eliminate cardiorespiratory and lactate variables and any measures derived directly from the study endpoints to prevent information leakage, while heart rate variables were not excluded. Variables with more than 30% missing examinations and athletes lacking complete CPX or body-composition profiles were discarded. After cleaning, 100 randomly selected single-measurement athletes served as an athlete-disjoint hold-out test set, withheld from model selection; the remainder were used for training and cross-validation. For the Achievement Score training set, when an athlete had several exams with the same best competition result, only the most recent was kept.

Every predictive model was encapsulated in a scikit-learn Pipeline that moved the data through three mandatory stages. First, a K-nearest-neighbours imputer (K = 5) filled in missing entries with the average of the five most similar athletes. Second, the feature matrix was scaled, with the choice between standard and robust scaling included in the hyperparameter search. Finally, the processed matrix was passed to one of six base estimators—XGBoost, random forest, histogram-based gradient boosting, multilayer perceptron (MLP), elastic-net, or support-vector regressor—or to one of two leakage-safe voting ensembles built over the three best-performing base pipelines (an unweighted mean vote, and a soft vote weighted by cross-validated R^2^). These algorithms provide complementary bias–variance profiles; the voting ensembles combine them without the intra-fold leakage of meta-learner stacking.

Hyperparameters were tuned with a randomized search of 1000 configurations per family combined with athlete-grouped five-fold cross-validation keyed on the de-identified Clinic number (GroupKFold for the Endurance Score; StratifiedGroupKFold for the Achievement Score, which also preserves class balance across folds). This grouping ensures that no athlete appears in both the training and validation portion of any fold, correcting an inflation of cross-validated R^2^ in our prior protocol caused by 259 athletes with repeated training exams. The primary metric was R^2^; mean absolute error (MAE) and root mean square error (RMSE) were auxiliary. The best-performing algorithm was retrained on the full training set. The final model for each endpoint maximized mean grouped-CV R^2^, with ties decided by the lower MAE; an ensemble was selected only when it outperformed every single-model candidate on the leakage-free cross-validation.

For hold-out validation, the entire pipeline, including imputation and scaling, was applied unchanged to the previously unseen test cohort; hold-out R^2^, MAE, and RMSE are reported with bootstrap 95% confidence intervals. Because the Achievement Score is an ordinal 1–5 outcome, hold-out performance was also summarized with rank and category-based metrics. Spearman’s rank correlation was computed between the continuous model predictions and the observed scores. Continuous predictions were then rounded to the nearest integer and clipped to the range 1–5. On these rounded categories we report quadratic-weighted Cohen’s kappa, exact-category accuracy, within-one-category accuracy model explainability was addressed with SHAP on the hold-out set. Sampling variability of the hold-out SHAP rankings was assessed by resampling the 100 hold-out athletes 1000 times and recomputing mean |SHAP| ranks in each resample. For tree-based learners we employed the exact TreeExplainer, whereas the model-agnostic KernelExplainer wrapped the MLP and the voting ensembles. SHAP furnishes several advantages. First, it delivers locally exact additive attributions that sum to the model prediction, allowing clinicians to understand precisely which exams contributed to an individual athlete’s Score being up or down. Second, the values are expressed in the target’s standard physical units, meaning that importances are directly comparable across algorithms and features. Third, every attribution carries a sign: positive SHAP values indicate factors that increase the predicted performance, whereas negative values indicate the opposite, providing immediate directional insight. Finally, aggregating SHAP values across the hold-out population produces global importance rankings and summary plots that highlight the features exerting the greatest influence on model behavior, thereby guiding future physiological data collection and intervention design.

### 2.6. Statistical Analyses

The source dataset comprised 2043 cardiopulmonary examinations from 885 competitive athletes. Observations were sequentially filtered to retain records that fulfilled the following criteria: availability of a valid CPX, complete anthropometric data (fat-free mass, body-mass index and SMM), CPX was considered maximal test (described in Section 2.1), and presence of all mandatory cardiopulmonary endpoints (VO_2max_, oxygen pulse, max VE, and HR at the first ventilatory threshold).

Duplicate examinations were removed, and biologically implausible values were detected with a modified median absolute deviation procedure. Records containing missing observations in analytic variables were discarded; imputation was not applied for the descriptive statistics. Missing values were imputed only inside the machine learning pipeline (Section 2.4).

Descriptive statistics: Normality of continuous variables was tested with the Shapiro–Wilk procedure (α = 0.05). Data exhibiting a Gaussian distribution are presented as mean ± standard deviation; skewed data are summarized as median and inter-quartile range. Categorical variables are expressed as absolute and relative frequencies.

Group comparisons: Binary comparisons between athlete groups (e.g., lower versus higher Achievement Score or Endurance Score strata) were performed with the Mann–Whitney U test for continuous outcomes owing to frequent departures from normality, and with Pearson’s χ^2^ test for categorical outcomes, defaulting to Fisher’s exact method when an expected cell count was below five. Effect sizes for categorical contrasts are reported as Cramér’s V.

Descriptive statistics, group comparisons and correlations were computed at the athlete level using one examination per operational athlete identity. The most recent CPX date was selected; ties were resolved by higher test number and then stable source order. Available-case denominators were reported for each variable, confidence intervals were obtained by bootstrap, and correlation analyses were labelled exploratory with Benjamini–Hochberg false-discovery-rate adjustment. Examination-level results and an earliest-examination sensitivity are provided in the Supplementary Material File S1.

Correlation analysis: Linear associations between numeric predictors and each of the two study outcomes, the Endurance Score and the Achievement Score, were quantified using Pearson correlation coefficients. Only variables with at least ten paired observations entered the analysis. Two-sided *p*-values are reported to three significant digits, or as <0.001 when appropriate.

Significance threshold and software environment: A two-sided *p* < 0.05 defined statistical significance. All analyses were implemented as a scripted Python 3 workflow rather than a standalone graphical application. Data handling used Pandas 2.2 and NumPy 1.26; conventional statistical tests used SciPy 1.17; and figures were produced with Matplotlib 3.8. Supervised learning, athlete-grouped cross-validation, imputation and scaling were implemented in scikit-learn 1.8, gradient-boosted trees were additionally fitted with XGBoost 3.2, and predictor attributions were obtained with the SHAP library. Fitted pipelines were stored with joblib.

## 3. Results

### 3.1. Study Design and Population

Between 2017 and 2024, a total of 885 elite athletes underwent detailed sports cardiology screening at our Clinic. We included only those athletes who were tested on the same equipment and completed all the tests. We excluded 197 athletes: 109 lacked body-composition analysis, 52 did not perform CPX, and 36 did not reach vita maxima load during CPX.

Our ML-based study included 688 healthy, asymptomatic elite athletes (19.7 ± 6.9 years, males: *n* = 477, 74.7%, training history: 12.0 ± 5.9 years, weekly training: 16.9 ± 7.1 hours) as a final study cohort who underwent 1194 sports cardiology screening exams. After the inclusion criteria, missingness among the 14 predictors did not exceed 5.8%; remaining missing values were imputed within each training fold by K-nearest neighbours. Each model used the same 14 predictors, giving 39 examinations (39 athletes) per predictor for the Achievement Score (551 examinations from 539 athletes) and 83 examinations (42 athletes) per predictor for the Endurance Score (1158 examinations from 588 athletes); the hold-out set provided 7 examinations per predictor. A random sample of 100 athletes who underwent 100 exams was split into a hold-out test set (21.5 ± 9.2 years, males *n* = 67, 67.0%, training history: 11.4 ± 4.9 years, weekly training: 16.1 ± 7.1 hours). The training population consisted of 588 athletes with 1094 examinations (21.4 ± 7.6 years; males, *n* = 410; 76.2% with training history: 12.1 ± 6.1 years; weekly training: 17.1 ± 7.2 hours). Most of them were members of the Hungarian National Teams (39.2%), academies (33.0%), and athletes from various sports clubs (27.8%). They were mainly swimmers (25.2%), basketball players (24.4%), football players (16.3%), water polo players (13.5%), and handball players (12.2%).

**Figure 2:**
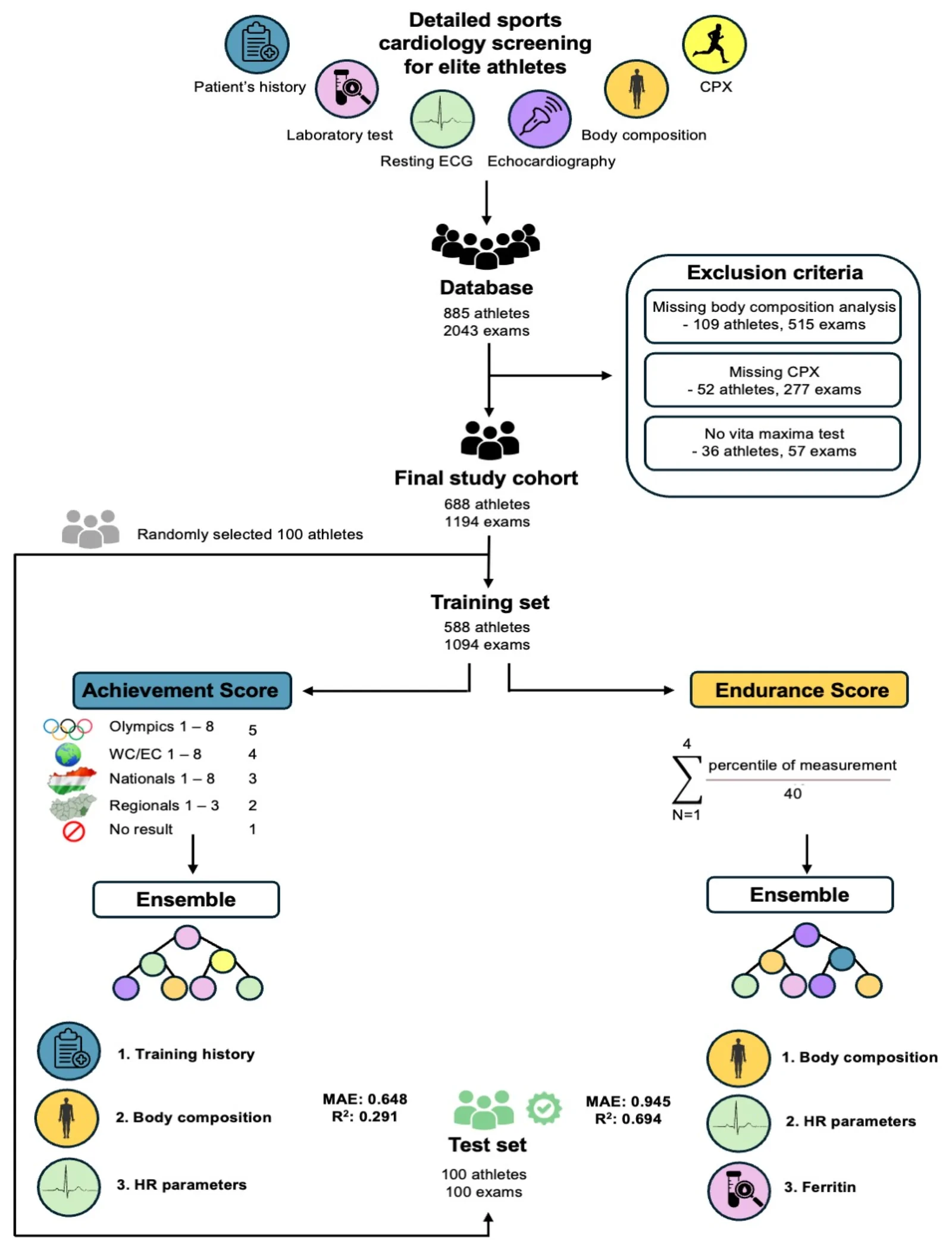
Study design and main results. All athletes underwent detailed sports cardiology screening, including a questionnaire on medical history, laboratory tests, resting ECG, echocardiography, body-composition analysis, and CPX. We established a database from the examined parameters. Athletes were excluded from the final study cohort because of missing body-composition analysis, CPX exams, or not reaching vita maxima load during CPX. From the final study cohort, we randomly selected 100 athletes as a hold-out test set. We created two scores, the Achievement and Endurance Scores, and with ML, we assessed the most important factors behind them. Regarding the Achievement Score, training history has the most significant impact, while body-composition parameters were identified as the most significant determinants of the Endurance Score. We calculated the results on the hold-out test set. Abbreviations: ECG: electrocardiogram, CPX: cardiopulmonary exercise test, WC: World Championship, EC: European Championship, HR: heart rate, MAE: mean absolute error, R^2^: coefficient of determination.

To comprehensively study sports performance, we established two scores: the Achievement Score and the Endurance Score. The Achievement Score represents the athlete’s best result, while the Endurance Score defines absolute cardiorespiratory fitness (Figure 2/Supplementary Material File S2, Table 1). The baseline characteristics of the cohorts are presented in Supplementary Material File S1.

### 3.2. Achievement Score

**Figure 3:**
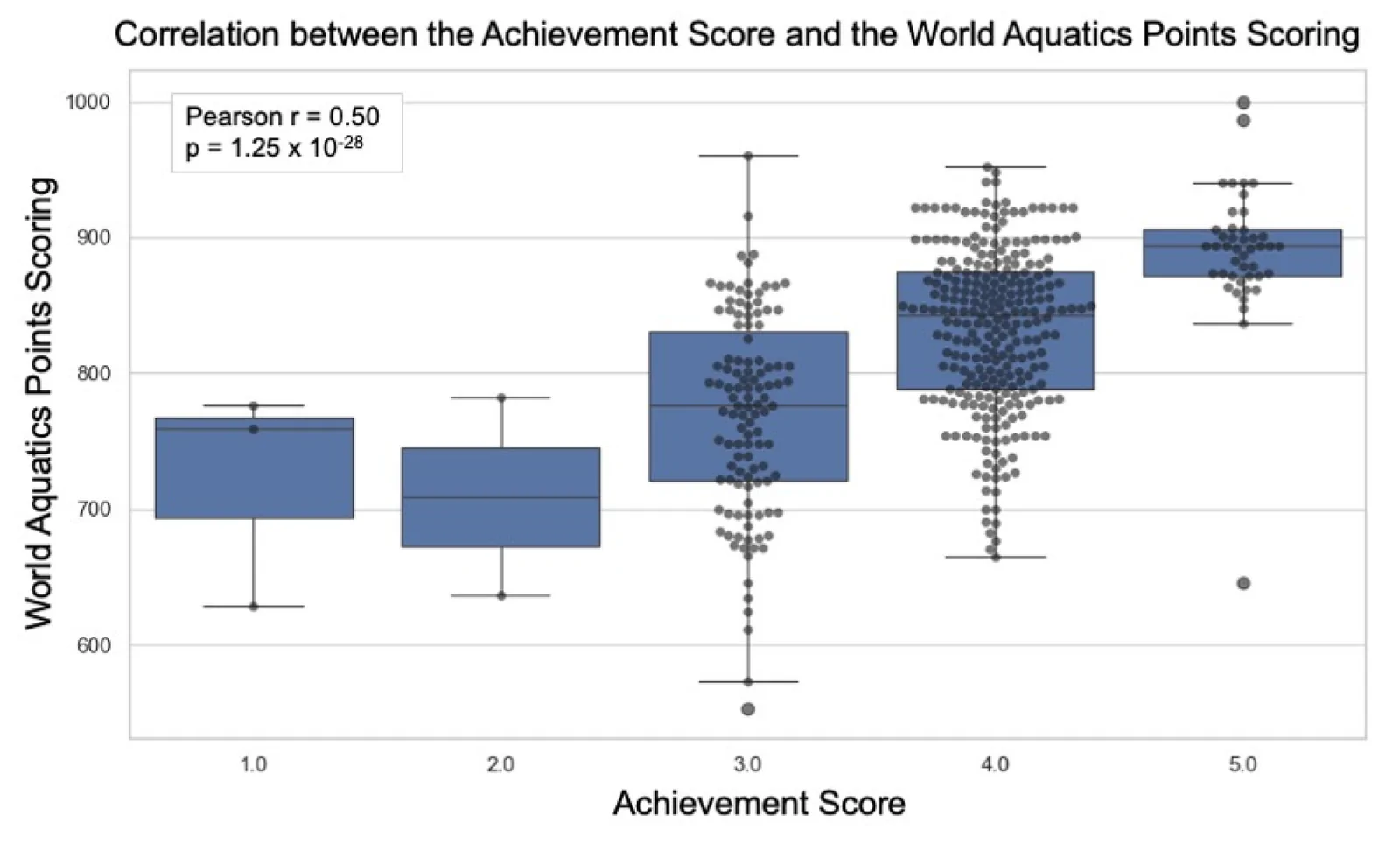
The correlation between the Achievement Score and the World Aquatics Points Scoring. The correlation between the Achievement Score and the World Aquatics Points Scoring was significant (Pearson r = 0.50, *p* < 0.05).

To validate the Achievement Score with the World Aquatics Points Scoring. The correlation between the Achievement Score and the World Aquatics Points Scoring was significant (Pearson r = 0.50, *p* < 0.05) (Figure 3).

**Figure 4:**
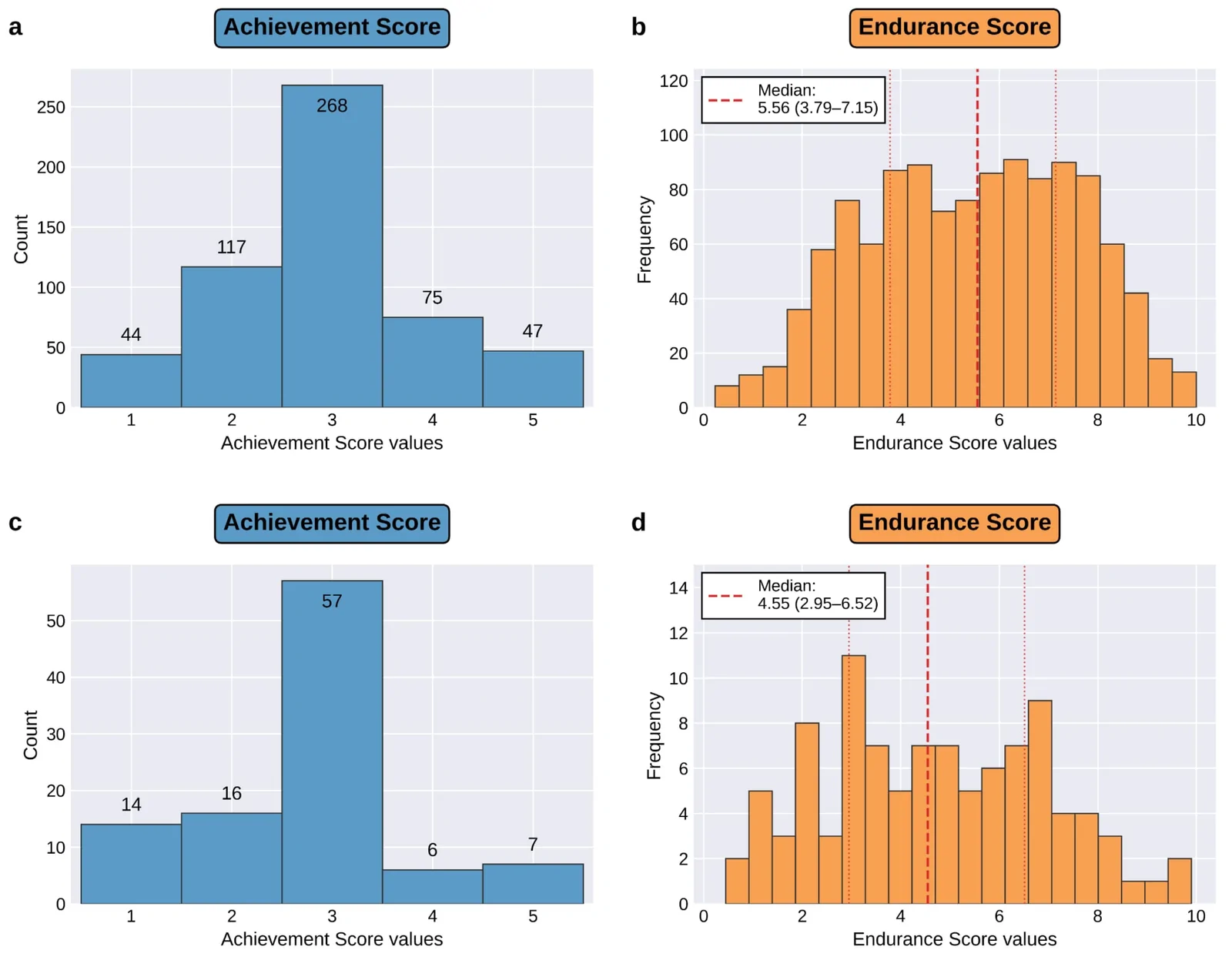
The distribution of the Achievement and Endurance Scores. Characteristics of the training set: (**a**,**b**). Characteristics of the hold-out test set: (**c**,**d**). (**a**,**c**): Regarding the Achievement Score, the majority of athletes received 3 points in both the training and hold-out test sets. (**b**,**d**): The distribution of the Endurance Score in both analyses.

The Achievement Score (1–5 points) was calculated based on the athlete’s best-achieved result closest in time to our examination (Figure 2/Supplementary Material File S2). We excluded 37 athletes from the analysis of the Achievement Score who had more than one examination with the same best competition result. Almost 10% of the athletes received a score of 5, which indicates that our population consisted of top-level athletes (Figure 4a). The athletes with scores above 3 were mainly swimmers (58%, mean Achievement Score: 3.8) and water polo players (29%, mean Achievement Score: 3.6).

**Figure 5:**
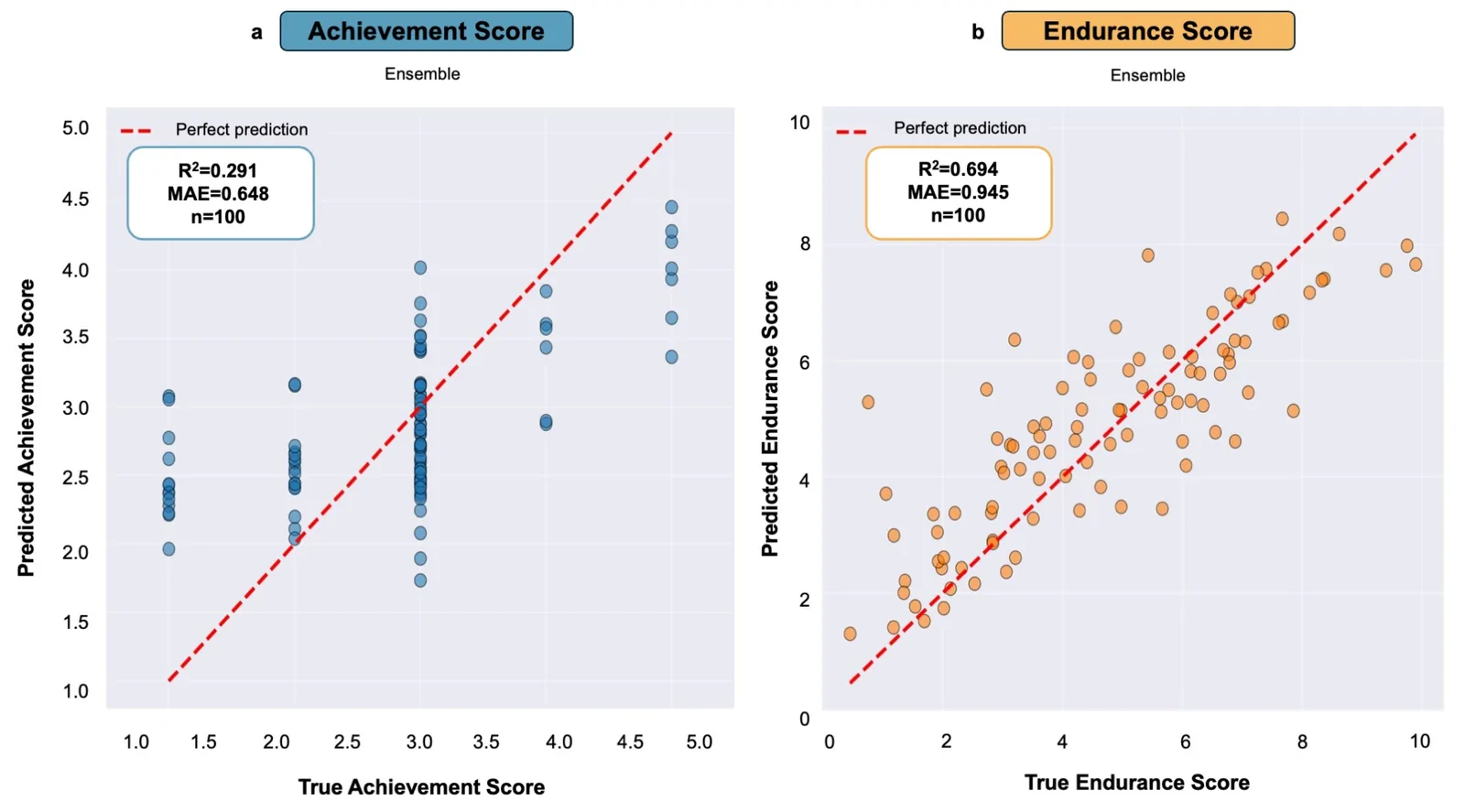
The results of the evaluation on a hold-out test set. (**a**) Evaluation on a hold-out test set of the Achievement Score; (**b**) Evaluation on a hold-out test set of the Endurance Score. As a hold-out test set, 100 athletes with 100 exams were randomly selected. For estimating the Achievement Score, a weighted voting ensemble of XGBoost, histogram-based gradient boosting and random forest performed best, whereas for the Endurance Score, an unweighted voting ensemble of XGBoost, histogram-based gradient boosting and a multilayer perceptron proved most effective. Achievement predictions were compressed toward the modal score of 3; after rounding, no hold-out case was assigned to class 1 or class 5. At higher values, there is a slight spread in both scores; however, our analysis remains reliable for R^2^, MAE, and RMSE. Abbreviations: R^2^: coefficient of determination, MAE: mean absolute error, RMSE: root mean square deviation.

The best-performing model for this endpoint was the R^2^-weighted voting ensemble of XGBoost, histogram-based gradient boosting and random forest, with a cross-validated R^2^ of 0.36 (95% CI (0.24, 0.48)) on the training set using athlete-grouped five-fold cross-validation, and 0.29 (95% CI (0.00, 0.489)) on the hold-out test set (Table 2, Figure 5). Spearman correlation was 0.528 (0.352–0.676), quadratic-weighted kappa was 0.473 (0.303 to 0.602), exact rounded-category accuracy was 47% (37% to 57%), and within-one-category accuracy was 94% (89% to 98%) on the hold-out test set.

The most important factors underlying the Achievement Score were training years and weekly training hours. Older athletes with more training years tended to have more experience and a higher chance of achieving better results throughout their careers. Regarding body-composition analysis, weight, PBF, height, and SMM had the greatest influence on the achieved results. Echocardiographic signs of sports adaptation also contributed to sports success, as larger left ventricular and left atrial sizes were observed in more successful athletes. (Figure 5).

According to the exercise test parameters, the most successful athletes achieved the highest treadmill times with a comparable proportion of anaerobic exercise. Additionally, they demonstrated a higher maximum oxygen pulse. The VO_2max_ values peaked in athletes who scored three and four points, similar to the maximum ventilation. Peak lactate was the highest in athletes with three points, but the lowest in athletes with the best achievements. Although the ejection fraction was comparable across all subgroups, the left ventricular end-systolic and end-diastolic diameters were increased in the more successful athletes. TAPSE showed insubstantial differences between the subgroups. While the levels of transferrin and soluble transferrin receptor were lower among the more successful athletes, transferrin saturation was higher in this group (Supplementary Material File S1—Table S1).

### 3.3. Endurance Score

The Endurance Score (0–10 points) is a multiparametric value that summarizes the results of CPX (Figure 1). The distribution of the Endurance Score suggests a balanced variation across individuals. The majority of athletes scored between 4 and 7 points, while only a small proportion were ranked in the 0–4 and 7–10 point ranges (Figure 4b).

The R^2^ was 0.68 (95% CI (0.64, 0.72)) on the training set using athlete-grouped five-fold cross-validation, and 0.69 (95% CI (0.56, 0.79)) for this endpoint in the hold-out test set validation analysis. The best-performing model was the unweighted voting ensemble of XGBoost, histogram-based gradient boosting and the multilayer perceptron, and the Endurance Score was predicted with a higher R^2^ than the Achievement Score (Table 2, Figure 5).

Based on the SHAP analysis, higher SMM, weight, PBF, peak HR, and HR reserve values were associated with higher model-predicted Endurance Scores. According to the resampled SHAP rankings three leading features were stable. Features ranked below fifth had overlapping importance intervals, and we do not interpret their order. Regarding the laboratory parameters, ferritin showed the strongest association with the model predictions for endurance. Longer training history, reflected by more weekly training hours and training years, was associated with higher predicted Achievement Scores. Older age was also associated with higher model-predicted Achievement Scores (Figure 5).

Athletes with higher Endurance Scores performed better during the exercise test, exhibiting greater treadmill time, longer aerobic exercise time, higher VO_2max_ values, increased ventilation, and higher oxygen pulse. Furthermore, their peak lactate levels were also higher than those of less-trained athletes. The ejection fraction values were in almost the same range across all score groups. The left ventricular end-systolic diameter and TAPSE values were higher in athletes with better physical fitness. Lower transferrin and soluble transferrin receptor values, along with elevated transferrin saturation, were observed in athletes with higher Endurance Scores (Supplementary Material File S1).

**Figure 6:**
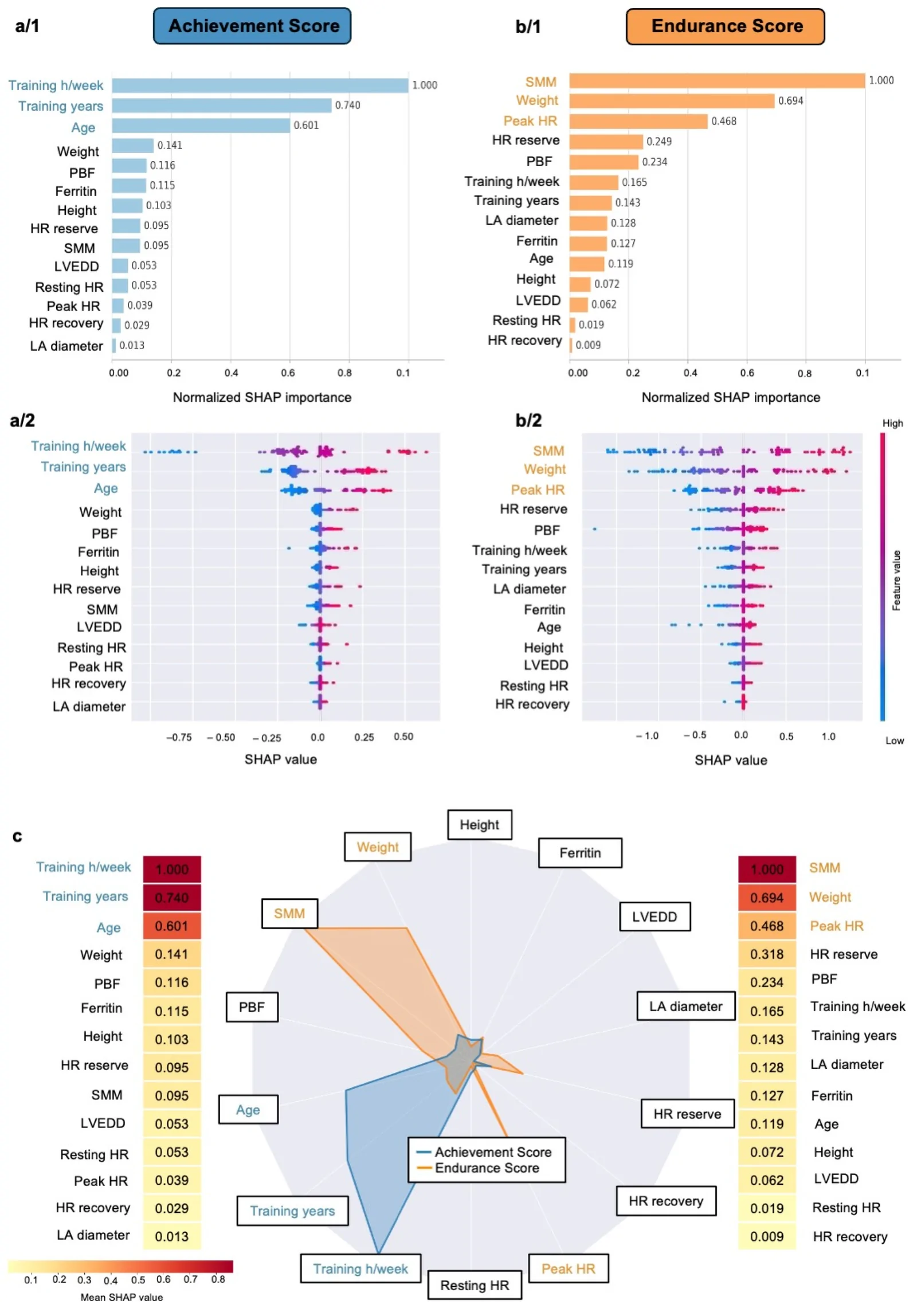
The main determinants behind the Achievement and Endurance Scores. Achievement Score feature importance with normalised SHAP values (**a/1**) and SHAP summary (**a/2**). For the Achievement Score, training history was the most important determinant, followed by body-composition and HR parameters. The exact SHAP values are in the figures. Endurance Score feature importance with normalised SHAP values (**b/1**) and SHAP (**b/2**) summary. Behind the Endurance Score, body-composition analysis and HR parameters were found as the main influencing factors. In (**a/2**) and (**b/2**), each dot is a test sample. Position shows impact direction. Colour shows feature value, blue represents lower, red represents higher values (normalised per feature). (**c**): The determinants behind the two scores are compared in a radar chart. The heatmap tables represent the SHAP values for both scores. SHAP values were computed on the hold-out set with KernelExplainer for the selected voting ensembles. Abbreviations: SMM: skeletal muscle mass (kg), HR: heart rate (bpm), LVEDD: left ventricular end-diastolic diameter (mm), LA: left atrium (mm), PBF: percentage body fat (%), SHAP: SHapley Additive exPlanations. Weight is measured in kilograms, height in centimetres, age in years, and ferritin level in microgram/L.

The key parameters affecting the two scores exhibit distinct patterns; the Achievement Score was predominantly influenced by the athlete’s experience, whereas the Endurance Score reflected body composition and sports adaptation. Although years of training and weekly training hours were more influential in determining achievements, SMM and body weight were the primary factors associated endurance. Among laboratory test parameters, ferritin was the most important, with a more substantial impact on endurance. Regarding HR parameters, peak HR was more critical for the Endurance Score, whereas resting HR was more relevant for the Achievement Score. Although echocardiography parameters are not the primary determinants for either score, they play a more substantial role in defining endurance (Figure 6). The Achievement and Endurance Scores demonstrate a correlation, whereby higher Achievement Score values are associated with increased Endurance Scores.

## 4. Discussion

Our study aimed to identify the most important determinants of sports performance during sports cardiology screening using ML. We assessed sports performance from two distinct perspectives: achievements and endurance, establishing the Achievement and Endurance Scores accordingly. The scores enabled the prediction and comparison of sports success and absolute physical fitness. Our main findings indicate that sports success was primarily affected by athletes’ experience, including their weekly training hours, training history, and age. In contrast, endurance was mainly influenced by body composition and sports adaptation, namely SMM, weight, and peak HR. Regarding laboratory parameters, ferritin emerged as the most significant determinant. Among echocardiographic parameters, left ventricular and left atrial diameters had the strongest association with sports performance. According to our results, the MAE values indicate that the predicted achievement and endurance scores differ from the true scores by approximately one score category on average. The R^2^ values describe the connection between the variables, which confirms a stronger connection regarding the Endurance Score.

Previous ML studies have mainly focused on predicting specific measures of exercise capacity, such as VO_2max_, using anthropometric, body-composition, and HR-related parameters [18]. In a study by Wenzel et al., both VO_2max_ and peak power output were predicted from non-exercise parameters. Body composition, particularly SMM, was identified as an important predictor of exercise capacity [19]. More recently, athletic performance has been assessed using a single composite performance score [20]. However, our study, by evaluating achievement and endurance separately, indicated that different sports cardiology screening parameters are relevant for predicting these two aspects of athletic performance.

The most important determinants of the Achievement Score in our study were weekly training hours, training years, and age, highlighting the relevance of both current training amount and accumulated experience. The development of sport-specific adaptations requires sufficient training exposure maintained over time. Therefore, both weekly training volume and training history may contribute to improved athletic performance [21,22]. Although training years and age are closely related, they may reflect different aspects of athletic development. Training years represent accumulated sport-specific experience, whereas age may also capture maturation and age-related changes in performance. The relationship between age and athletic performance is generally curvilinear, and the age of peak performance varies considerably across sports [23,24,25,26]. In technical disciplines, accumulated experience and decision-making skills may favor peak performance at older ages, whereas in sports such as swimming, early specialization may provide younger athletes with a competitive advantage [26].

The main determinants of the Endurance Score in our study were SMM, weight, and peak HR. The importance of SMM in our model is consistent with previous evidence supporting the central role of skeletal muscle in endurance performance [27]. Skeletal muscle is the primary site of oxygen utilization during exercise, and its oxidative capacity, capillarization, fiber composition, and oxygen extraction ability contribute to aerobic performance [27]. Greater SMM may provide a larger peripheral capacity for oxygen utilization and energy production during exercise. Nevertheless, endurance performance depends not only on muscle mass but also on the metabolic and functional characteristics of skeletal muscle [27].

Body weight was also an important predictor of the Endurance Score, with higher values associated with better endurance. This finding should be interpreted together with the importance of SMM, as greater body weight in athletes may reflect greater muscle mass. Furthermore, because the Endurance Score represents absolute exercise capacity, greater metabolically active mass may partly explain the observed association. However, the relationship between body weight and athletic performance is likely to vary according to the characteristics of the different sports [28].

Peak HR was also identified as an important predictor of the Endurance Score. This finding may be interpreted in the context of the role of HR in the cardiovascular response to exercise. According to the Fick principle, oxygen uptake depends partly on cardiac output, which is determined by HR and stroke volume [29]. An appropriate chronotropic response during maximal exercise contributes to increased cardiac output, thereby supporting oxygen delivery to exercising skeletal muscles. Therefore, the importance of peak HR in our model may reflect the integrated cardiovascular response required to sustain high levels of exercise performance.

Iron is essential for oxygen transport and muscle function through its involvement in hemoglobin and myoglobin and also contributes to mitochondrial electron transport, oxidative metabolism, and energy production [30]. Although ferritin is not directly involved in these processes, it reflects the body’s iron stores and may indicate iron depletion before the development of anemia [31]. Inadequate iron stores may impair oxygen delivery and utilization during exercise, which may partly explain the predictive importance of ferritin, particularly for the Endurance Score. Several studies have reported improved endurance performance following iron supplementation in iron-depleted individuals [32,33,34]. However, other studies found that improvements in iron status were not accompanied by increases in VO_2max_ or endurance performance, particularly in non-anemic athletes [13,35]. Therefore, our findings indicate that ferritin has predictive relevance but do not imply that higher ferritin levels necessarily enhance athletic performance.

Regarding the echocardiographic parameters, left ventricular and left atrial diameters were the most important predictors of sports performance, with greater diameters associated particularly with a higher Endurance Score. These findings may reflect the cardiac remodeling that develops in response to long-term training. Left ventricular dilatation is frequently observed in the athlete’s heart, particularly in athletes participating in high-dynamic sports. A larger left ventricular chamber can accommodate a greater end-diastolic volume and may thereby increase stroke volume and cardiac output during prolonged intensive exercise [36]. Similarly, increased left atrial diameter may reflect the long-term hemodynamic demands associated with increased ventricular filling and sustained cardiac output. Consistent with this, a meta-analysis by Iskandar et al. demonstrated larger left atrial dimensions in elite athletes than in non-athletic controls [37]. Thus, the predictive importance of left ventricular and left atrial diameters in our models may reflect cumulative cardiovascular adaptation to long-term training, particularly in relation to endurance performance.

Our study can help athletes to better understand the underlying factors of sports performance. We study the Achievement and the Endurance Score separately; therefore, athletes may have a better picture of the meaning of their results. The Endurance Score simplifies the results of the CPX measurement into a single point and represents and ranks the cardiorespiratory fitness of the athletes. Our predictions can provide feedback to athletes, coaches, and team members about athletes’ training status and endurance, and may help them improve their preparation and individual development.

### Strengths and Limitations

A strength of this study is the application of artificial intelligence to the assessment of sports performance, a complex and multidimensional construct that cannot be adequately captured by any single conventional measure. The use of machine learning models enables the quantification of complex multivariable interactions and the integration of multiple physiological and performance-related factors. While AI is commonly used to accelerate or validate existing analytical approaches and support decision-making, its application in this context offers a novel approach to characterizing a construct for which no single established assessment method exists.

Another important strength is the large clinical dataset underlying the AI analyses, comprising comprehensive sports cardiology screening data, including CPX, from a substantial cohort of elite athletes. Although AI-based approaches to sports performance have been reported previously, few have been supported by similarly extensive clinical data combined with objective competitive outcomes. The inclusion of a large number of high-level athletes together with their competition rankings further strengthens the clinical and performance relevance of the Achievement Score.

Our study has some limitations. It is a cross-sectional single center study, which may limit the interpretation and applicability of our findings in a broader aspect. The inclusion of elite athletes enhances the reproducibility in elite levels; however, it needs to be tested in amateur and master athletes for general use. With our cross-sectional design, we cannot establish training effects, a longitudinal study is needed to assess training-induced adaptations.

The Achievement Score also has some additional limitations, as the ranking category may not reflect an equivalent level of competitive performance across different sports. Differences in the number of participants, competitive structure, and depth of competition may influence the significance of a given ranking position. Consequently, the 1–5 achievement scale should be interpreted as a standardized approximation of competitive success rather than a directly comparable measure of performance across sporting disciplines. Swimming was selected to validate the Achievement Score due to its substantial representation and objective, time-based performance measures. However, restricting the sport-specific analysis to swimmers may limit the generalizability of our findings to other sporting disciplines.

Another limitation is that performance was measured in a laboratory environment, which differs from the athletes’ sport-specific own environment. CPXs were performed on a treadmill ergometer, which in some sports may not reflect the nature of the movements. In our study, we were focused on endurance and CPX, however strengths, speed, power or agility are also important determinants. Future studies are needed to assess their role and significance in the context of sport performance.

## Figures and Tables

**Table 1:** Comparison of the different study cohorts.

	Final Study Cohort *n* = 639 (AS)/688 (ES)	Test Set *n* = 100	Training Set *n* = 539 (AS)/588 (ES)	*p* Value
Achievement Score	1.0:57;	1.0:14;	1.0:43;	0.049
	2.0:127;	2.0:16;	2.0:111;	
	3.0:322;	3.0:57;	3.0:265;	
	4.0:79;	4.0:6;	4.0:73;	
	5.0:54	5.0:7	5.0:47	
Endurance Score	5.4 (3.5–7.1)	4.5 (3.0–6.5)	5.6 (3.7–7.1)	0.004
Age (years)	18.0 (16.0–23.0)	18.0 (15.0–24.2)	18.0 (16.0–23.0)	0.874
Training years	11.0 (8.0–15.0)	10.0 (8.0–15.0)	11.0 (8.0–15.0)	0.283
Training h/week	18.0 (12.5–20.0)	15.0 (10.0–20.0)	20.0 (14.0–20.0)	0.001
Resting HR (1/min)	67.0 (60.0–76.0)	67.0 (60.0–77.2)	67.0 (59.0–76.0)	0.528
Peak HR (1/min)	193.0 (187.0–200.0)	193.0 (184.0–200.0)	193.0 (187.0–200.0)	0.326
HR recovery (1/min)	27.0 (21.0–32.0)	27.0 (19.0–31.0)	27.0 (21.0–32.0)	0.515
HR reserve (1/min)	120.0 (112.0–128.0)	119.5 (108.0–128.0)	120.0 (112.0–128.0)	0.370
Treadmill time (min)	13.0 (11.0–14.0)	12.0 (10.6–14.0)	13.0 (11.1–14.0)	0.046
Anaerobic threshold VO_2_ (%)	0.3 (0.3–0.4)	0.3 (0.2–0.4)	0.3 (0.3–0.4)	0.538
Anaerobic threshold HR (1/min)	176.0 (169.0–184.0)	178.5 (167.8–185.5)	176.0 (169.0–184.0)	0.640
VO_2max_ (L/min)	4.0 (3.3–4.5)	3.6 (3.0–4.2)	4.0 (3.4–4.5)	<0.001
VO_2max_ (mL/kg/min)	53.4 (48.3–57.3)	50.2 (45.6–55.9)	53.8 (48.8–57.4)	0.001
Max ventilation (L/min)	142.5 ± 29.1	134.4 ± 30.7	144.0 ± 28.6	0.002
O_2_ pulse max (mL/beat)	20.7 (17.4–23.8)	18.8 (16.2–22.4)	21.0 (17.7–23.9)	0.001
Peak lactate (mmol/L)	9.5 (7.7–11.3)	9.3 (7.3–11.3)	9.6 (7.8–11.4)	0.257
Height (cm)	182.0 (175.0–188.0)	178.7 (173.0–185.9)	182.0 (175.0–189.0)	0.007
Weight (kg)	74.7 (65.0–82.0)	70.0 (63.4–79.6)	75.0 (65.8–82.9)	0.028
SMM (kg)	37.1 (31.6–41.9)	34.7 (30.0–40.0)	37.7 (32.0–42.1)	0.013
PBF (%)	11.9 (8.8–16.7)	13.5 (10.4–18.5)	11.6 (8.7–16.1)	0.013
EF (%)	60.0 (58.0–65.0)	60.0 (59.0–65.0)	60.0 (58.0–65.0)	0.398
LA diameter (mm)	37.4 ± 5.2	37.2 ± 5.3	37.4 ± 5.2	0.536
LVESD (mm)	33.0 (30.0–36.0)	33.0 (29.0–36.0)	33.0 (30.0–36.0)	0.330
LVEDD (mm)	48.0 (45.0–51.0)	47.0 (44.0–51.0)	48.0 (45.0–51.0)	0.162
TAPSE (mm)	24.0 (21.0–27.0)	24.0 (21.0–27.0)	24.0 (21.0–27.0)	0.840
Ferritin (ug/L)	60.0 (36.0–94.0)	60.0 (30.2–97.8)	60.0 (37.0–93.8)	0.786
Transferrin (g/L)	2.7 (2.5–3.0)	2.8 (2.5–3.1)	2.7 (2.4–3.0)	0.022
Transferrin saturation (%)	27.0 (19.2–35.0)	26.0 (19.0–36.0)	27.0 (20.0–35.0)	0.775
Soluble transferrin R (mg/L)	3.0 (2.5–3.7)	3.0 (2.7–3.7)	3.0 (2.4–3.7)	0.289

Values are median (interquartile range) or mean ± standard deviation when the Shapiro–Wilk test indicated normality in the whole sample and in the test and training subsets. The Achievement Score is shown as category counts (1–5). *p* values compare the hold-out with the training set (Mann–Whitney U for continuous variables; χ^2^ for the Achievement Score). One examination per athlete was selected: the most recent CPX date, with ties broken by higher test number and then stable source order. Header denominators are endpoint-specific (Achievement/Endurance). Cells other than the Endurance Score use athletes with both scores (overall *n* = 639, training *n* = 539, hold-out *n* = 100); the Endurance Score row uses the Endurance-eligible athlete set (overall *n* = 688, training *n* = 588, hold-out *n* = 100). Abbreviations: AS: Achievement Score, ES: Endurance Score, h: hours, HR: heart rate, VO_2_ max: maximum oxygen consumption, max: maximum, O_2_: oxygen, SMM: skeletal muscle mass, PBF: percentage body fat, EF: ejection fraction, LA: left atrium, LVESD: left ventricular end-systolic diameter, LVEDD: left ventricular end-diastolic diameter, TAPSE: tricuspid annular plane systolic excursion, R: receptor.

**Table 2:** Athlete-grouped five-fold cross-validation performance of the eight candidate models for predicting Achievement Score and Endurance Score in the training set. For each endpoint, models are ranked by descending mean cross-validated R^2^ (Rank = 1 denotes the best-performing model). “Train score” refers to performance on the training folds, whereas “Cross-validation score” refers to performance on the corresponding validation folds within athlete-grouped five-fold cross-validation. The table reports the mean R^2^ and MAE across the five folds ± standard deviation. MAE is expressed in Score points, so values closer to zero indicate lower prediction error. The best model for the Achievement Score endpoint was an R^2^-weighted voting ensemble of XGBoost, histogram-based gradient boosting and random forest; the best model for the Endurance Score endpoint was an unweighted voting ensemble of XGBoost, histogram-based gradient boosting and a multilayer perceptron. The hyperparameters of the ensemble components are given in Appendix A.

	Model	Rank	Train Score		Cross-Validation Score	
			Mean R^2^	Mean MAE	Mean R^2^	Mean MAE
Achievement Score	Weighted voting ensemble	1	0.59 *	–	0.36 ± 0.08	–
	Unweighted voting ensemble	2	–	–	0.36 ± 0.08	–
	XGBoost	3	0.50 ± 0.02	0.55 ± 0.01	0.36 ± 0.09	0.62 ± 0.04
	Histogram-based gradient boosting	4	0.62 ± 0.05	0.48 ± 0.03	0.35 ± 0.09	0.63 ± 0.04
	Random forest	5	0.61 ± 0.01	0.49 ± 0.01	0.34 ± 0.07	0.63 ± 0.03
	Support-vector regressor	6	0.55 ± 0.01	0.47 ± 0.01	0.29 ± 0.06	0.65 ± 0.04
	Elastic net	7	0.31 ± 0.03	0.64 ± 0.01	0.28 ± 0.10	0.65 ± 0.06
	MLP	8	0.35 ± 0.04	0.62 ± 0.02	0.26 ± 0.09	0.66 ± 0.04
Endurance Score	Unweighted voting ensemble	1	0.77 *	–	0.68 ± 0.03	–
	Weighted voting ensemble	2	–	–	0.68 ± 0.03	–
	XGBoost	3	0.81 ± 0.00	0.72 ± 0.01	0.66 ± 0.03	0.96 ± 0.04
	Histogram-based gradient boosting	4	0.79 ± 0.01	0.76 ± 0.02	0.66 ± 0.03	0.96 ± 0.03
	MLP	5	0.70 ± 0.01	0.91 ± 0.02	0.66 ± 0.02	0.97 ± 0.01
	Random forest	6	0.81 ± 0.01	0.70 ± 0.01	0.66 ± 0.04	0.97 ± 0.04
	Support-vector regressor	7	0.73 ± 0.01	0.83 ± 0.01	0.65 ± 0.03	0.99 ± 0.03
	Elastic net	8	0.66 ± 0.01	0.98 ± 0.01	0.64 ± 0.02	1.00 ± 0.04

Abbreviations: XGBoost: extreme gradient boosting, MLP: multilayer perceptron, R^2^: coefficient of determination, MAE: mean absolute error. * Single fitted value on the full training set; no per-fold spread is available for the voting ensembles. – Not applicable: per-fold MAE was not retained for the voting ensembles.

## Data Availability

The datasets presented in this study are available on request from the corresponding author. Due to patient data, privacy data are not made available publicly.

## Supplementary Materials

The following supporting information can be downloaded at https://www.mdpi.com/article/10.3390/sports14100431/s1. Supplementary Material File S1. Table S1: Baseline characteristics of the final study cohort (athlete-level), Table S2: Baseline characteristics of the training set (athlete-level), Table S3: Baseline characteristics of the test set (athlete-level). Supplementary Material File S2. Figure S1: Study design and main results.

## Abbreviations

The following abbreviations are used in this manuscript:

VO_2max_	maximum oxygen uptake
CPX	cardiopulmonary exercise testing
AI	artificial intelligence
ECG	electrocardiogram
HR	heart rate
SMM	skeletal muscle mass
PBF	percentage body fat
TAPSE	tricuspid annular plane systolic excursion
VE	ventilation
VT	tidal volume
VE/VCO_2_	the ratio of minute ventilation to carbon dioxide production
RER	respiratory exchange ratio
ML	machine learning
SHAP	SHapley Additive exPlanations
XGBoost	eXtreme Gradient Boosting
R^2^	coefficient of determination
RMSE	root mean square error
EF	ejection fraction
LA	left atrium
LVESD	left ventricular end-systolic diameter
LVEDD	left ventricular end-diastolic diameter
R	receptor
WC	World Championship
EC	European Championship
MLP	multilayer perceptron

## Appendix A. Hyperparameters of the Ensemble Components

**Table A1:** Achievement Score—R^2^-weighted voting ensemble. Abbreviations: XGBoost: extreme gradient boosting.

Component	Hyperparameters
XGBoost	colsample_bytree = 0.51, gamma = 4.46, learning_rate = 0.110, max_depth = 3, min_child_weight = 7, n_estimators = 389, reg_alpha = 0.220, reg_lambda = 1.14, subsample = 0.71
Histogram-based gradient boosting	l2_regularization = 2.21, learning_rate = 0.073, max_features = 0.72, max_iter = 460, max_leaf_nodes = 7, min_samples_leaf = 14
Random forest	bootstrap = True, max_depth = 6, max_features = 0.5, max_samples = 0.75, min_samples_leaf = 4, min_samples_split = 8, n_estimators = 405

**Table A2:** Endurance Score—unweighted voting ensemble. Abbreviations: XGBoost: extreme gradient boosting, MLP: multilayer perceptron.

Component	Hyperparameters
XGBoost	colsample_bytree = 0.64, gamma = 4.99, learning_rate = 0.016, max_depth = 6, min_child_weight = 3, n_estimators = 944, reg_alpha = 0.895, reg_lambda = 3.65, subsample = 0.68
Histogram-based gradient boosting	l2_regularization = 1.00, learning_rate = 0.049, max_features = 0.51, max_iter = 311, max_leaf_nodes = 9, min_samples_leaf = 60
MLP	hidden_layer_sizes = (5, 3), alpha = 0.379, learning_rate_init = 0.084, solver = ‘adam’, beta_1 = 0.914, beta_2 = 0.993
