# Supplementary material for: Machine Learning-Based Estimation of Sports Performance in Elite Athletes
Source: Sports (Basel). 2026 Oct 4;14(10):431. doi: 10.3390/sports14100431 (PMC13709816; doi:10.3390/sports14100431)
Supplement: Supplementary file 1 [file sports-14-00431-s001.zip › Supplementary Material File S1.pdf]

Table S1. Baseline characteristics of the final study cohort (athlete-level).

	All records	AS = 1, n=57	AS = 2, n=127	AS = 3, n=322	AS = 4, n=79	AS = 5, n=54	AS correl	AS p value	ES (0-2(n=38	ES (2-4(n=153	ES (4-6(n=158	ES (6-8(n=215	ES (8-10) n=75	ES correl	ES p value
**Achievement Score**	1.0 : 57;<br>2.0 : 127;<br>3.0 : 322;<br>4.0 : 79;<br>5.0 : 54	1.0 : 57	2.0 : 127	3.0 : 322	4.0 : 79	5.0 : 54	1.000	<0.001	1.0 : 10;<br>2.0 : 8;<br>3.0 : 14;<br>4.0 : 4;<br>5.0 : 2	1.0 : 19;<br>2.0 : 22;<br>3.0 : 74;<br>4.0 : 26;<br>5.0 : 12	1.0 : 18;<br>2.0 : 41;<br>3.0 : 77;<br>4.0 : 12;<br>5.0 : 10	1.0 : 8;<br>2.0 : 45;<br>3.0 : 119;<br>4.0 : 22;<br>5.0 : 21	1.0 : 2;<br>2.0 : 11;<br>3.0 : 38;<br>4.0 : 15;<br>5.0 : 9	0.134	<0.001
**Endurance Score**	5.6 (3.5-7.1)	4.0 (2.5-5.3)	5.6 (4.1-6.8)	5.9 (3.7-7.1)	5.7 (3.1-7.5)	6.3 (3.8-7.7)	0.134	<0.001	1.3 (0.8-1.8)	3.0 (2.6-3.4)	5.0 (4.5-5.5)	6.9 (6.4-7.4)	8.5 (8.2-9.0)	1.000	<0.001
**Age**	18.0 (16.0-23.0)	17.0 (14.0-26.0)	16.0 (15.0-18.0)	18.0 (16.0-22.0)	22.0 (18.0-25.0)	27.0 (21.2-30.0)	0.372	<0.001	19.0 (16.0-24.8)	17.0 (15.0-23.0)	17.0 (15.0-22.0)	19.0 (16.0-24.0)	19.0 (18.0-22.0)	0.139	<0.001
**Training years**	11.0 (8.0-15.0)	9.0 (6.5-12.0)	8.0 (7.0-10.0)	11.0 (8.0-15.0)	14.0 (12.0-17.0)	17.5 (14.0-22.0)	0.466	<0.001	9.0 (6.0-12.0)	10.5 (8.0-14.0)	10.0 (7.0-13.0)	12.0 (9.0-16.8)	12.0 (10.0-15.5)	0.180	<0.001
**Training h/week**	18.0 (12.5-20.0)	10.0 (8.0-15.0)	15.0 (10.5-20.0)	16.5 (13.5-20.0)	21.0 (20.0-25.0)	25.0 (20.0-28.0)	0.433	<0.001	14.0 (10.0-20.0)	20.0 (13.0-22.0)	15.0 (10.9-20.0)	20.0 (14.0-20.0)	20.0 (16.0-20.0)	0.121	0.002
**Resting HR**	67.0 (60.0-76.0)	72.0 (64.0-80.0)	71.0 (62.0-78.0)	67.5 (59.2-75.8)	63.0 (57.0-71.0)	63.0 (56.0-70.0)	-0.204	<0.001	69.0 (61.0-78.5)	70.0 (61.0-78.0)	68.0 (61.0-76.0)	65.0 (58.0-73.0)	64.5 (58.0-74.0)	-0.163	<0.001
**Peak HR**	193.0 (187.0-200.0)	196.0 (187.0-203.0)	196.0 (190.0-200.0)	193.0 (187.0-200.0)	193.0 (183.0-201.0)	187.0 (181.0-195.2)	-0.126	0.001	187.0 (176.8-193.0)	196.0 (187.0-200.0)	196.0 (187.0-203.0)	193.0 (187.0-200.0)	193.0 (190.0-200.0)	0.054	0.173
**HR recovery**	27.0 (21.0-32.0)	27.0 (20.0-32.0)	27.0 (21.0-32.0)	27.0 (21.0-32.0)	24.0 (20.0-30.0)	27.0 (22.0-33.0)	-0.030	0.455	27.0 (20.0-32.0)	27.0 (22.0-33.0)	27.0 (22.0-32.0)	26.0 (20.0-31.0)	24.0 (20.0-30.0)	-0.085	0.033
**HR reserve**	120.0 (112.0-128.0)	117.0 (104.0-128.0)	118.5 (112.2-127.0)	121.0 (113.0-128.0)	120.0 (110.8-131.2)	120.0 (108.2-127.8)	0.052	0.188	112.5 (103.2-122.8)	118.0 (109.0-126.0)	120.5 (112.0-128.2)	120.0 (113.0-128.0)	125.0 (120.0-131.0)	0.212	<0.001
**Treadmill time**	13.0 (11.0-14.0)	12.0 (9.0-13.5)	12.0 (11.0-13.8)	13.0 (11.0-14.0)	13.0 (11.9-15.0)	14.0 (12.7-15.0)	0.232	<0.001	10.0 (8.4-11.5)	12.0 (10.5-14.0)	12.5 (11.0-14.0)	13.0 (12.0-14.5)	14.0 (13.0-15.0)	0.389	<0.001
**Anaerobic%**	0.3 (0.3-0.4)	0.3 (0.3-0.4)	0.4 (0.2-0.5)	0.3 (0.3-0.4)	0.3 (0.2-0.4)	0.3 (0.2-0.4)	-0.116	0.003	0.4 (0.3-0.5)	0.3 (0.3-0.4)	0.4 (0.3-0.4)	0.3 (0.3-0.4)	0.3 (0.2-0.3)	-0.174	<0.001
**Anaerobic threshold HR**	176.0 (169.0-184.0)	176.0 (166.0-184.0)	179.0 (171.5-187.0)	176.0 (170.0-184.0)	171.0 (163.0-183.5)	172.5 (166.0-180.0)	-0.114	0.004	166.0 (160.0-172.0)	179.0 (169.0-184.0)	177.5 (171.0-187.0)	175.0 (168.5-184.0)	179.0 (171.0-187.0)	0.093	0.019
**$VO_{2\,max}$ (l/min)**	4.0 (3.3-4.5)	3.4 (2.9-4.0)	3.9 (3.4-4.3)	4.0 (3.4-4.5)	4.0 (3.3-4.8)	4.3 (3.5-4.8)	0.170	<0.001	2.6 (2.3-2.8)	3.1 (2.9-3.4)	3.8 (3.5-4.0)	4.4 (4.2-4.6)	5.0 (4.8-5.3)	0.938	<0.001
**$VO_{2\,max}$ (ml/kg/min)**	53.4 (48.3-57.3)	49.4 (43.5-54.7)	54.0 (48.7-56.5)	53.8 (49.2-57.5)	53.0 (48.8-58.1)	51.3 (46.5-57.8)	0.082	0.039	43.1 (39.4-49.3)	48.4 (44.7-53.2)	52.6 (48.7-56.1)	55.8 (51.7-58.2)	57.6 (54.7-60.0)	0.535	<0.001
**Max ventilation**	143.0 (121.5-161.0)	127.0 (104.0-149.0)	142.0 (127.0-156.0)	144.0 (123.0-161.0)	141.0 (118.5-172.5)	145.5 (124.2-172.0)	0.118	0.003	97.5 (89.0-104.0)	115.0 (106.0-124.0)	138.0 (128.0-148.8)	158.0 (147.0-168.5)	183.0 (171.0-193.0)	0.885	<0.001
**O_2 pulse max**	20.7 (17.4-23.8)	17.2 (15.8-21.2)	20.2 (17.8-22.6)	20.8 (17.8-23.8)	22.1 (17.2-26.0)	23.0 (18.6-26.5)	0.193	<0.001	14.1 (12.8-16.3)	16.4 (14.7-18.2)	19.7 (18.1-21.4)	23.3 (21.6-25.1)	26.1 (24.4-28.1)	0.862	<0.001
**Peak lactate**	9.5 (7.7-11.3)	9.4 (7.4-11.1)	9.2 (7.8-11.4)	9.9 (8.0-11.5)	9.4 (7.3-11.5)	8.8 (7.5-9.9)	-0.034	0.395	7.8 (6.7-9.8)	9.1 (7.4-10.8)	9.5 (7.7-11.1)	9.9 (8.3-11.8)	10.0 (8.2-11.8)	0.162	<0.001
**Height**	182.0 (175.0-188.0)	177.3 (172.0-183.0)	184.0 (176.5-190.0)	182.0 (175.0-187.0)	180.0 (171.2-188.0)	185.0 (177.0-192.0)	0.049	0.216	167.0 (162.1-172.1)	173.0 (167.7-178.0)	180.0 (176.2-186.0)	186.0 (181.2-191.0)	189.0 (184.0-193.4)	0.660	<0.001
**Weight**	74.7 (65.0-82.0)	68.5 (63.0-80.0)	74.4 (65.3-81.7)	75.0 (66.0-81.0)	73.0 (63.0-85.9)	82.0 (67.9-94.0)	0.119	0.003	57.2 (53.1-65.5)	64.3 (58.5-69.4)	73.0 (66.2-78.0)	80.0 (74.0-85.0)	86.0 (82.0-93.6)	0.682	<0.001
**SMM**	37.1 (31.6-41.9)	33.5 (28.9-37.0)	37.1 (33.2-41.8)	37.9 (32.2-41.5)	36.1 (28.6-43.8)	41.3 (32.3-45.2)	0.091	0.021	25.9 (23.7-29.7)	29.9 (27.1-33.5)	35.9 (33.2-39.2)	40.9 (38.0-43.7)	43.8 (41.1-47.0)	0.741	<0.001
**PBF**	11.9 (8.8-16.7)	14.1 (10.4-19.4)	10.7 (7.5-14.1)	11.4 (8.7-15.8)	13.8 (11.0-17.8)	14.7 (11.2-19.3)	0.132	<0.001	18.5 (13.6-20.9)	15.7 (12.3-20.4)	11.3 (8.0-15.6)	10.4 (8.4-12.8)	11.4 (9.7-14.3)	-0.334	<0.001

**EF**	60.0 (58.0-65.0)	60.0 (59.0-65.0)	60.0 (56.2-65.0)	60.0 (58.0-65.0)	60.0 (56.8-65.0)	60.0 (57.8-63.5)	-0.003	0.949	60.0 (60.0-63.5)	60.0 (59.0-65.0)	60.0 (58.0-65.0)	60.0 (58.0-65.0)	60.0 (55.0-63.0)	-0.127	0.002
**LA diameter**	37.0 (34.0-41.0)	36.0 (32.0-42.0)	37.0 (33.0-40.0)	37.0 (34.0-41.0)	37.0 (34.0-43.0)	38.5 (36.0-41.0)	0.104	0.011	34.0 (30.8-37.2)	35.0 (33.0-40.0)	36.0 (33.5-40.0)	38.0 (35.0-41.0)	40.0 (36.0-43.0)	0.278	<0.001
**LVESD**	33.0 (30.0-36.0)	30.0 (28.0-35.0)	33.0 (30.0-35.0)	33.0 (30.0-36.0)	33.0 (31.0-37.0)	34.0 (30.0-36.0)	0.108	0.008	30.0 (27.0-31.0)	31.0 (28.0-34.0)	33.0 (30.0-35.0)	35.0 (31.0-37.0)	36.0 (33.0-39.0)	0.418	<0.001
**LVEDD**	48.0 (45.0-51.0)	47.0 (41.0-51.0)	48.0 (44.8-50.0)	48.0 (45.0-51.0)	49.0 (45.0-51.0)	51.0 (47.0-53.0)	0.166	<0.001	45.0 (41.8-47.0)	45.0 (42.0-49.0)	48.0 (45.5-51.0)	49.0 (47.0-52.0)	51.0 (48.0-55.0)	0.414	<0.001
**TAPSE**	24.0 (21.0-27.0)	24.0 (22.0-27.0)	24.0 (21.0-27.0)	24.0 (21.0-27.0)	24.0 (21.0-26.0)	24.0 (21.0-27.0)	-0.028	0.493	24.0 (20.8-27.0)	24.0 (21.0-26.0)	23.0 (20.0-27.0)	24.0 (22.0-27.0)	25.0 (21.5-28.5)	0.084	0.040
**Ferritin**	60.0 (36.0-94.0)	44.5 (20.0-77.2)	53.0 (37.0-80.0)	62.0 (37.0-99.0)	59.5 (39.2-93.8)	87.5 (51.0-119.8)	0.148	<0.001	32.0 (21.2-69.8)	43.0 (26.0-68.0)	58.0 (32.0-92.0)	70.0 (51.0-105.0)	77.0 (57.0-112.0)	0.351	<0.001
**Transferrin**	2.7 (2.5-3.0)	2.9 (2.6-3.2)	2.7 (2.5-2.9)	2.7 (2.4-3.0)	2.8 (2.4-3.1)	2.7 (2.4-2.9)	-0.055	0.169	2.8 (2.6-3.1)	2.9 (2.6-3.1)	2.7 (2.5-3.0)	2.6 (2.4-2.9)	2.6 (2.4-2.9)	-0.208	<0.001
**Transferrin saturation**	27.0 (19.2-35.0)	24.5 (19.8-30.2)	25.0 (19.0-32.2)	28.5 (20.0-35.2)	27.0 (18.0-37.5)	29.0 (22.0-36.0)	0.092	0.024	19.5 (15.5-28.2)	25.5 (18.8-36.0)	26.0 (18.0-37.0)	29.0 (22.0-35.0)	29.5 (24.0-36.5)	0.172	<0.001
**Soluble transferrin R**	3.0 (2.5-3.7)	3.2 (2.9-3.8)	3.1 (2.6-3.8)	3.0 (2.4-3.6)	3.1 (2.5-3.6)	2.8 (2.5-3.4)	-0.095	0.024	3.3 (2.7-4.2)	3.0 (2.5-3.6)	3.2 (2.6-3.8)	3.0 (2.4-3.6)	3.0 (2.4-3.5)	-0.093	0.027

n=639 athletes (one most recent examination per raw clinic-number identity among records with both scores). Continuous variables are median (IQR) unless Shapiro–Wilk normality held in the whole sample and in every Achievement and Endurance stratum and its complement, in which case mean ± SD is shown. Correlations are Spearman rank coefficients (exploratory).

Abbreviations: AS: Achievement Score, ES: Endurance Score, h: hours, HR: heart rate, VO2 max: maximum oxygen consumption, max: maximum, O2: oxygen, SMM: skeletal muscle mass, PBF: percentage body fat, EF: ejection fraction, LA: left atrium, LVESD: left ventricle end-systolic diameter, LVEDD: left ventricle end-diastolic diameter, TAPSE: tricuspid annular plane systolic excursion, R: receptor.

Table S2. Baseline characteristics of the training set (athlete-level).

	All records	AS = 1, n=43	AS = 2, n=111	AS = 3, n=265	AS = 4, n=73	AS = 5, n=47	AS correl	AS p value	ES (0-2(n=25	ES (2-4(n=125	ES (4-6(n=132	ES (6-8(n=189	ES (8-10) n=68	ES correl	ES p value
**Achievement Score**	1.0 : 43; 2.0 : 111; 3.0 : 265; 4.0 : 73; 5.0 : 47	1.0 : 43	2.0 : 111	3.0 : 265	4.0 : 73	5.0 : 47	1.000	<0.001	1.0 : 5; 2.0 : 4; 3.0 : 10; 4.0 : 4; 5.0 : 2	1.0 : 15; 2.0 : 19; 3.0 : 60; 4.0 : 24; 5.0 : 7	1.0 : 14; 2.0 : 35; 3.0 : 63; 4.0 : 10; 5.0 : 10	1.0 : 8; 2.0 : 42; 3.0 : 98; 4.0 : 21; 5.0 : 20	1.0 : 1; 2.0 : 11; 3.0 : 34; 4.0 : 14; 5.0 : 8	0.114	0.008
**Endurance Score**	5.8 (3.7-7.3)	4.2 (2.7-5.6)	5.9 (4.3-7.0)	5.9 (3.9-7.2)	5.8 (3.1-7.6)	6.8 ű(4.8-7.8)	0.114	0.008	1.3 (0.8-1.7)	3.0 (2.6-3.4)	5.0 (4.5-5.5)	6.9 (6.4-7.5)	8.5 (8.2-8.9)	1.000	<0.001
**Age**	18.0 (16.0-23.0)	17.0 (15.0-22.0)	16.0 (15.0-18.0)	18.0 (16.0-22.0)	22.0 (18.0-25.0)	27.0 (21.5-30.0)	0.414	<0.001	19.0 (16.0-23.0)	17.0 (15.0-23.0)	17.0 (15.0-22.0)	19.0 (16.0-24.0)	19.0 (17.8-22.0)	0.166	<0.001
**Training years**	11.0 (8.0-15.0)	9.0 (6.0-12.0)	8.0 (7.0-10.0)	11.0 (9.0-15.0)	14.0 (12.0-17.0)	18.0 (14.0-23.0)	0.489	<0.001	9.5 (5.8-12.2)	10.0 (8.0-14.0)	10.0 (7.0-13.5)	12.0 (9.0-17.0)	12.0 (10.0-15.0)	0.172	<0.001
**Training h/week**	20.0 (14.0-20.0)	10.2 (9.0-15.0)	18.0 (12.0-20.0)	18.0 (13.5-20.0)	22.0 (20.0-25.0)	25.0 (20.0-27.8)	0.417	<0.001	16.0 (11.5-20.0)	20.0 (13.5-21.0)	15.0 (12.0-20.0)	20.0 (14.0-20.0)	20.0 (16.0-20.0)	0.106	0.014
**Resting HR**	67.0 (59.0-76.0)	72.0 (63.5-80.5)	71.0 (62.0-78.0)	68.0 (59.0-75.0)	63.0 (57.0-72.5)	63.5 (56.0-70.8)	-0.190	<0.001	69.0 (61.0-74.0)	71.0 (62.0-78.0)	67.0 (60.0-76.0)	65.0 (58.0-74.0)	64.0 (58.0-74.0)	-0.155	<0.001
**Peak HR**	193.0 (187.0-200.0)	196.0 (187.0-203.0)	196.0 (190.0-200.0)	193.0 (187.0-200.0)	193.0 (184.0-203.0)	190.0 (182.5-196.0)	-0.137	0.001	187.0 (176.0-193.0)	194.0 (190.0-200.0)	196.0 (187.0-200.8)	193.0 (187.0-200.0)	194.5 (190.0-200.5)	0.040	0.356
**HR recovery**	27.0 (21.0-32.0)	27.0 (18.5-32.5)	27.0 (22.0-32.0)	27.0 (21.0-32.0)	24.0 (20.0-30.0)	27.0 (20.5-33.0)	-0.039	0.367	23.5 (19.8-33.0)	27.0 (22.0-34.0)	27.0 (22.0-32.8)	26.0 (20.0-31.5)	24.5 (20.0-30.0)	-0.082	0.060
**HR reserve**	120.0 (112.0-128.0)	118.0 (105.0-128.0)	119.0 (113.0-127.0)	121.0 (112.0-127.0)	120.0 (111.2-131.8)	121.0 (111.0-129.0)	0.038	0.380	114.0 (110.0-125.0)	117.0 (111.0-126.0)	121.0 (113.0-129.0)	120.0 (113.0-127.5)	125.0 (120.0-131.5)	0.182	<0.001
**Treadmill time**	13.0 (11.1-14.0)	12.0 (9.7-13.2)	12.5 (11.0-14.0)	13.0 (11.2-14.0)	13.0 (12.0-15.0)	14.0 (12.8-14.8)	0.221	<0.001	10.0 (9.0-11.5)	12.0 (10.5-14.0)	12.5 (11.0-14.0)	13.0 (12.0-14.5)	14.0 (13.0-15.0)	0.388	<0.001
**Anaerobic%**	0.3 (0.3-0.4)	0.4 (0.3-0.5)	0.3 (0.2-0.5)	0.3 (0.3-0.4)	0.3 (0.2-0.4)	0.3 (0.3-0.4)	-0.121	0.005	0.4 (0.3-0.5)	0.4 (0.3-0.4)	0.3 (0.3-0.4)	0.3 (0.3-0.4)	0.3 (0.2-0.4)	-0.190	<0.001
**Anaerobic threshold HR**	176.0 (169.0-184.0)	176.0 (168.5-184.0)	179.0 (172.0-187.0)	176.0 (170.0-184.0)	172.0 (163.0-183.0)	173.0 (165.0-180.5)	-0.122	0.005	166.0 (160.0-171.0)	178.0 (169.0-184.0)	176.0 (171.0-187.0)	175.0 (169.0-184.0)	179.5 (171.0-187.0)	0.098	0.022
**VO_{2max} (l/min)**	4.0 (3.4-4.5)	3.4 (3.1-4.1)	4.0 (3.6-4.4)	4.0 (3.4-4.5)	4.0 (3.3-4.8)	4.4 (3.7-4.8)	0.152	<0.001	2.5 (2.4-2.8)	3.2 (2.9-3.4)	3.8 (3.5-4.0)	4.4 (4.2-4.6)	5.0 (4.7-5.3)	0.935	<0.001
**VO_{2max} (ml/kg/min)**	53.8 (48.8-57.4)	50.4 (44.6-55.5)	54.2 (49.9-56.5)	53.9 (49.4-57.5)	53.7 (48.9-58.2)	53.7 (47.2-58.0)	0.068	0.114	42.5 (39.3-49.4)	49.3 (45.2-53.7)	52.8 (48.9-56.3)	55.7 (51.7-58.1)	57.8 (55.5-60.3)	0.518	<0.001
**Max ventilation**	145.0 (123.0-163.0)	137.0 (110.0-150.0)	146.0 (129.5-158.0)	145.0 (124.0-161.0)	144.0 (119.0-172.0)	148.0 (129.0-175.0)	0.086	0.047	99.0 (89.0-104.0)	117.0 (107.0-125.0)	138.5 (128.0-148.2)	158.0 (147.0-169.0)	181.0 (170.8-191.2)	0.879	<0.001
**O_2 pulse max**	21.0 (17.7-23.9)	18.2 (15.9-21.5)	20.8 (18.2-22.8)	21.2 (18.2-23.9)	22.1 (17.2-26.0)	23.5 (18.9-26.6)	0.180	<0.001	13.8 (12.8-16.0)	16.5 (14.9-18.4)	19.7 (18.3-21.4)	23.4 (21.7-25.1)	25.9 (24.2-28.1)	0.858	<0.001
**Peak lactate**	9.6 (7.8-11.4)	9.6 (8.1-11.0)	9.3 (7.7-11.5)	10.0 (8.1-11.4)	9.4 (7.5-11.6)	8.8 (7.5-10.0)	-0.038	0.379	7.9 (7.0-10.8)	9.1 (7.5-10.7)	9.5 (7.8-11.0)	9.9 (8.4-11.8)	10.0 (8.2-11.8)	0.150	<0.001
**Height**	182.0 (175.0-189.0)	178.0 (172.0-182.5)	185.0 (177.4-190.0)	182.0 (176.0-187.4)	180.0 (170.0-188.0)	185.0 (176.0-192.0)	0.012	0.774	168.0 (162.0-172.0)	173.0 (167.0-179.0)	180.8 (176.0-186.0)	186.0 (181.0-191.0)	189.0 (184.4-193.9)	0.647	<0.001
**Weight**	75.0 (65.8-82.9)	71.0 (63.5-81.0)	74.5 (67.0-82.0)	76.0 (66.0-81.0)	73.0 (62.0-88.0)	83.0 (69.9-94.0)	0.106	0.013	57.0 (52.0-64.0)	64.3 (58.0-69.9)	73.0 (66.3-77.5)	80.0 (75.0-85.0)	85.5 (81.7-93.0)	0.685	<0.001
**SMM**	37.7 (32.0-42.1)	34.5 (30.0-37.4)	37.5 (34.2-41.8)	38.3 (32.5-41.6)	34.9 (28.3-43.9)	41.4 (33.0-45.4)	0.079	0.068	25.5 (23.2-29.8)	29.7 (26.9-33.6)	35.9 (33.2-39.2)	41.2 (38.3-43.7)	43.8 (41.0-46.6)	0.743	<0.001
**PBF**	11.6 (8.7-16.1)	14.1 (10.3-17.0)	10.3 (7.4-13.4)	11.3 (8.5-15.4)	14.1 (11.1-17.9)	14.6 (10.4-18.4)	0.170	<0.001	17.0 (13.5-20.1)	15.0 (11.8-20.3)	11.3 (8.2-16.7)	10.3 (8.2-12.8)	11.4 (9.6-14.3)	-0.306	<0.001

**EF**	60.0 (58.0-65.0)	60.0 (58.8-61.5)	60.0 (56.0-65.0)	60.0 (58.0-65.0)	60.0 (57.2-65.0)	60.0 (57.0-63.0)	0.019	0.676	60.0 (60.0-63.2)	60.0 (58.0-65.0)	60.0 (57.0-65.0)	60.0 (58.0-65.0)	60.0 (55.0-63.5)	-0.101	0.023
**LA diameter**	37.4 ± 5.2	36.7 ± 5.6	36.8 ± 5.2	37.4 ± 5.1	38.3 ± 5.3	38.0 ± 4.9	0.088	0.048	32.5 ± 4.3	35.9 ± 4.9	37.0 ± 5.4	38.5 ± 4.6	39.8 ± 4.9	0.327	<0.001
**LVESD**	33.0 (30.0-36.0)	31.5 (28.2-35.8)	33.0 (30.0-36.0)	33.0 (30.0-36.0)	33.0 (30.5-37.0)	34.0 (30.0-36.0)	0.057	0.203	29.0 (27.0-31.0)	31.0 (28.0-34.0)	33.0 (30.0-35.0)	34.0 (31.0-37.0)	35.0 (33.0-39.0)	0.408	<0.001
**LVEDD**	48.0 (45.0-51.0)	47.5 (40.8-51.0)	48.0 (45.0-50.0)	48.0 (45.0-51.5)	49.0 (45.0-51.0)	51.0 (47.0-53.0)	0.128	0.004	45.0 (42.8-47.0)	46.0 (42.0-49.0)	49.0 (45.5-51.0)	49.0 (47.0-52.0)	51.0 (48.0-55.0)	0.393	<0.001
**TAPSE**	24.0 (21.0-27.0)	24.0 (22.0-26.5)	23.5 (21.0-27.0)	24.0 (21.0-27.0)	24.0 (21.0-26.0)	24.0 (21.0-26.0)	-0.021	0.636	24.0 (20.0-27.2)	24.0 (21.0-26.0)	23.0 (20.0-26.8)	24.0 (22.0-27.0)	25.0 (21.0-28.0)	0.085	0.056
**Ferritin**	60.0 (37.0-93.8)	46.0 (20.0-80.8)	53.0 (36.2-78.5)	65.0 (38.0-100.0)	58.5 (33.8-96.2)	90.0 (57.0-121.0)	0.151	<0.001	29.0 (21.0-47.0)	43.5 (25.0-69.2)	54.0 (32.5-91.5)	70.5 (51.0-105.2)	77.0 (59.2-111.8)	0.367	<0.001
**Transferrin**	2.7 (2.4-3.0)	2.9 (2.6-3.2)	2.7 (2.5-2.9)	2.6 (2.4-3.0)	2.8 (2.4-3.1)	2.7 (2.4-2.8)	-0.048	0.274	2.8 (2.6-3.0)	2.8 (2.5-3.1)	2.7 (2.4-3.0)	2.6 (2.4-2.9)	2.6 (2.4-2.9)	-0.167	<0.001
**Transferrin saturation**	27.0 (20.0-35.0)	24.5 (20.8-31.2)	25.0 (19.0-32.0)	29.0 (20.0-35.8)	27.0 (18.0-36.8)	28.5 (22.0-35.2)	0.068	0.123	20.5 (15.5-29.2)	25.0 (18.0-35.0)	26.0 (19.0-36.8)	29.0 (23.0-35.0)	29.0 (24.0-36.0)	0.160	<0.001
**Soluble transferrin R**	3.0 (2.4-3.7)	3.2 (2.7-3.8)	3.1 (2.5-3.8)	2.9 (2.3-3.6)	3.1 (2.5-3.6)	2.8 (2.5-3.4)	-0.084	0.064	3.8 (2.7-4.4)	3.0 (2.5-3.5)	3.2 (2.5-3.8)	2.9 (2.4-3.6)	3.0 (2.1-3.5)	-0.084	0.063

n=539 athletes (one most recent examination per raw clinic-number identity among records with both scores). Continuous variables are median (IQR) unless Shapiro–Wilk normality held in the whole sample and in every Achievement and Endurance stratum and its complement, in which case mean ± SD is shown. Correlations are Spearman rank coefficients (exploratory).

Abbreviations: AS: Achievement Score, ES: Endurance Score, h: hours, HR: heart rate, VO2 max: maximum oxygen consumption, max: maximum, O2: oxygen, SMM: skeletal muscle mass, PBF: percentage body fat, EF: ejection fraction, LA: left atrium, LVESD: left ventricle end-systolic diameter, LVEDD: left ventricle end-diastolic diameter, TAPSE: tricuspid annular plane systolic excursion, R: receptor.

Table S3. Baseline characteristics of the test set (athlete-level).

	All records	AS = 1,<br>n=14	AS = 2,<br>n=16	AS = 3,<br>n=57	AS = 4,<br>n=6	AS = 5,<br>n=7	AS correl	AS p value	ES (0-2(<br>n=13	ES (2-4(<br>n=28	ES (4-6(<br>n=26	ES (6-8(<br>n=26	ES (8-10)<br>n=7	ES correl	ES p value
**Achievement Score**	1.0 : 14;<br>2.0 : 16;<br>3.0 : 57;<br>4.0 : 6;<br>5.0 : 7	1.0 : 14	2.0 : 16	3.0 : 57	4.0 : 6	5.0 : 7	1.000	<0.001	1.0 : 5;<br>2.0 : 4;<br>3.0 : 4	1.0 : 4;<br>2.0 : 3;<br>3.0 : 14;<br>4.0 : 2;<br>5.0 : 5	1.0 : 4;<br>2.0 : 6;<br>3.0 : 14;<br>4.0 : 2	2.0 : 3;<br>3.0 : 21;<br>4.0 : 1;<br>5.0 : 1	1.0 : 1;<br>3.0 : 4;<br>4.0 : 1;<br>5.0 : 1	0.242	0.015
**Endurance Score**	4.5<br>(3.0-6.5)	3.2<br>(1.2-4.9)	4.1<br>(2.8-4.5)	5.7<br>(3.2-6.8)	5.0<br>(3.7-6.4)	3.2<br>(3.0-5.2)	0.242	0.015	1.4<br>(1.2-1.8)	3.0<br>(2.8-3.5)	5.0<br>(4.4-5.4)	6.8<br>(6.4-7.1)	8.6<br>(8.4-9.6)	1.000	<0.001
**Age**	18.0<br>(15.0-24.2)	22.5<br>(14.0-36.5)	15.5<br>(15.0-19.2)	18.0<br>(16.0-21.0)	23.5<br>(19.2-24.8)	29.0<br>(24.0-29.5)	0.160	0.112	19.0<br>(14.0-38.0)	19.0<br>(15.0-25.8)	17.0<br>(15.0-22.8)	18.5<br>(16.0-23.0)	20.0<br>(18.0-25.0)	-0.012	0.908
**Training years**	10.0<br>(8.0-15.0)	8.5<br>(7.0-13.2)	8.5<br>(7.0-11.2)	10.0<br>(8.0-13.5)	15.0<br>(14.0-18.0)	17.0<br>(15.0-17.5)	0.324	0.001	7.0<br>(6.5-7.5)	11.0<br>(9.2-17.0)	9.0<br>(7.2-11.8)	12.0<br>(10.0-15.0)	13.0<br>(10.5-17.5)	0.206	0.045
**Training h/week**	15.0<br>(10.0-20.0)	10.0<br>(8.0-12.5)	10.0<br>(10.0-16.2)	15.0<br>(13.0-20.0)	20.0<br>(20.0-21.5)	25.0<br>(23.2-30.2)	0.520	<0.001	10.0<br>(8.8-13.9)	18.0<br>(10.0-22.1)	14.0<br>(10.0-20.0)	15.0<br>(13.1-20.0)	18.0<br>(15.0-20.0)	0.167	0.099
**Resting HR**	67.0<br>(60.0-77.2)	71.5<br>(65.5-77.8)	69.5<br>(64.5-89.5)	67.0<br>(60.0-78.0)	64.5<br>(60.0-66.8)	56.0<br>(50.0-63.0)	-0.279	0.005	71.0<br>(61.0-87.0)	68.0<br>(60.0-76.8)	68.5<br>(65.0-78.8)	63.5<br>(57.8-70.8)	65.0<br>(58.0-72.5)	-0.199	0.047
**Peak HR**	193.0<br>(184.0-200.0)	194.5<br>(181.0-202.2)	191.5<br>(184.0-195.5)	195.0<br>(190.0-203.0)	184.5<br>(179.0-197.5)	181.0<br>(177.5-185.5)	-0.084	0.404	190.0<br>(179.0-193.0)	196.0<br>(186.2-203.0)	194.5<br>(181.8-203.0)	191.5<br>(184.8-202.2)	190.0<br>(188.5-198.0)	0.112	0.265
**HR recovery**	27.0<br>(19.0-31.0)	28.0<br>(25.5-32.0)	27.0<br>(19.0-30.2)	26.0<br>(19.0-31.0)	23.5<br>(22.2-31.5)	30.0<br>(28.5-35.5)	0.000	0.998	29.0<br>(27.0-31.0)	27.0<br>(19.0-32.0)	27.0<br>(19.8-30.8)	26.5<br>(19.0-30.8)	24.0<br>(21.0-26.5)	-0.127	0.209
**HR reserve**	118.3 ± 14.1	114.3 ± 17.2	113.2 ± 17.1	121.1 ± 11.9	116.2 ± 16.4	116.6 ± 13.4	0.132	0.190	108.7 ± 15.1	116.3 ± 13.6	118.3 ± 14.4	122.9 ± 12.7	126.9 ± 8.3	0.350	<0.001
**Treadmill time**	12.0<br>(10.6-14.0)	10.9<br>(7.0-13.4)	12.0<br>(10.4-12.3)	12.0<br>(11.0-14.0)	11.8<br>(10.2-13.6)	16.0<br>(12.2-17.8)	0.267	0.008	9.8<br>(6.8-10.5)	12.0<br>(11.2-14.0)	12.0<br>(10.6-13.0)	13.8<br>(12.0-14.9)	14.0<br>(12.5-15.2)	0.359	<0.001
**Anaerobic%**	0.3<br>(0.2-0.4)	0.3<br>(0.3-0.3)	0.4<br>(0.3-0.4)	0.3<br>(0.3-0.4)	0.3<br>(0.2-0.4)	0.2<br>(0.2-0.3)	-0.104	0.302	0.4<br>(0.3-0.4)	0.3<br>(0.2-0.3)	0.4<br>(0.3-0.4)	0.3<br>(0.2-0.4)	0.2<br>(0.2-0.3)	-0.112	0.267
**Anaerobic threshold HR**	178.5<br>(167.8-185.5)	178.0<br>(161.5-188.8)	179.5<br>(165.0-184.8)	179.0<br>(171.0-187.0)	168.5<br>(154.5-180.2)	171.0<br>(168.0-175.0)	-0.072	0.476	166.0<br>(160.0-176.0)	182.0<br>(176.8-187.0)	181.0<br>(166.0-184.8)	174.5<br>(168.5-187.0)	171.0<br>(170.0-184.0)	0.115	0.254
**$VO_{2\,max}$ (l/min)**	3.6<br>(3.0-4.2)	3.0<br>(2.7-3.7)	3.3<br>(2.9-3.7)	3.9<br>(3.3-4.3)	3.8<br>(3.4-4.6)	3.3<br>(3.1-3.9)	0.262	0.008	2.6<br>(2.3-2.8)	3.0<br>(2.7-3.3)	3.7<br>(3.5-3.9)	4.3<br>(4.2-4.5)	5.4<br>(5.1-5.5)	0.939	<0.001
**$VO_{2\,max}$ (ml/kg/min)**	50.2<br>(45.6-55.9)	47.2<br>(43.0-49.7)	47.0<br>(45.4-54.1)	52.5<br>(47.4-57.5)	51.4<br>(48.8-54.0)	48.4<br>(40.0-49.2)	0.138	0.172	43.5<br>(40.8-49.0)	47.5<br>(42.7-48.9)	52.1<br>(46.3-53.5)	57.2<br>(52.8-59.9)	54.5<br>(52.5-56.0)	0.590	<0.001
**Max ventilation**	133.0<br>(111.8-154.5)	107.5<br>(91.2-131.2)	125.0<br>(111.2-137.0)	143.0<br>(120.0-158.0)	136.0<br>(119.0-174.8)	132.0<br>(113.0-150.5)	0.278	0.005	92.0<br>(88.0-102.0)	113.0<br>(103.5-121.2)	134.5<br>(128.0-150.0)	156.0<br>(148.0-164.2)	191.0<br>(185.0-196.5)	0.904	<0.001
**O_2 pulse max**	18.8<br>(16.2-22.4)	16.5<br>(14.2-17.8)	17.5<br>(15.8-20.0)	20.2<br>(16.8-22.7)	21.7<br>(18.6-25.8)	18.6<br>(16.6-22.1)	0.262	0.008	14.3<br>(13.1-16.5)	15.9<br>(14.1-17.5)	19.4<br>(17.8-21.9)	22.5<br>(21.0-24.9)	26.9<br>(26.5-28.5)	0.859	<0.001
**Peak lactate**	9.3<br>(7.3-11.3)	7.7<br>(6.7-11.6)	8.9<br>(8.8-9.9)	9.6<br>(7.3-11.6)	7.2<br>(6.3-9.6)	8.5<br>(8.1-9.3)	-0.025	0.805	7.7<br>(6.5-8.8)	8.7<br>(7.1-11.7)	9.8<br>(7.9-11.3)	9.7<br>(7.6-12.1)	10.5<br>(8.8-10.9)	0.213	0.033
**Height**	178.4 ± 9.8	175.0 ± 10.2	175.9 ± 11.3	178.9 ± 9.5	182.2 ± 8.0	184.1 ± 7.4	0.241	0.016	166.6 ± 6.8	173.4 ± 7.8	181.4 ± 7.0	184.2 ± 8.3	188.0 ± 5.1	0.691	<0.001
**Weight**	70.0<br>(63.4-79.6)	63.9<br>(59.7-74.8)	65.5<br>(60.0-76.6)	71.0<br>(67.0-79.0)	73.7<br>(70.2-79.1)	68.5<br>(66.3-91.8)	0.228	0.023	58.4<br>(55.0-67.3)	66.0<br>(61.2-69.0)	72.0<br>(66.7-80.9)	76.1<br>(69.3-83.2)	95.8<br>(88.0-100.5)	0.633	<0.001
**SMM**	34.7<br>(30.0-40.0)	30.1<br>(26.8-36.0)	32.9<br>(29.8-37.8)	35.5<br>(31.4-40.0)	37.5<br>(34.9-40.1)	32.5<br>(31.4-42.3)	0.194	0.054	26.4<br>(24.0-29.4)	31.2<br>(27.7-32.6)	35.8<br>(33.3-38.4)	38.8<br>(35.0-42.4)	47.3<br>(44.0-50.6)	0.716	<0.001
**PBF**	13.5<br>(10.4-18.5)	18.5<br>(12.1-20.5)	13.8<br>(8.3-20.6)	12.9<br>(10.1-17.0)	11.3<br>(10.7-13.7)	18.9<br>(14.9-20.6)	-0.064	0.532	20.3<br>(18.3-21.2)	18.4<br>(14.1-20.5)	11.1<br>(7.9-14.1)	11.1<br>(10.1-13.1)	11.9<br>(11.2-16.8)	-0.454	<0.001

**EF**	60.0 (59.0-65.0)	60.0 (60.0-65.0)	60.0 (60.0-63.0)	60.0 (59.0-65.0)	59.5 (53.8-60.8)	62.0 (60.0-63.5)	-0.120	0.250	60.0 (60.0-64.0)	61.0 (60.0-65.0)	60.0 (60.0-65.0)	60.0 (57.0-65.0)	59.0 (55.0-60.0)	-0.247	0.016
**LA diameter**	37.0 (33.0-41.0)	35.0 (31.0-42.0)	36.5 (35.0-39.8)	35.0 (33.0-40.0)	39.0 (34.2-45.2)	41.0 (39.5-46.0)	0.165	0.111	40.0 (32.0-42.0)	36.0 (33.8-40.2)	35.5 (33.0-39.2)	38.0 (34.0-40.0)	39.0 (33.5-43.0)	-0.004	0.968
**LVESD**	33.0 (29.0-36.0)	29.0 (27.0-30.0)	30.0 (27.0-33.2)	34.0 (31.0-37.0)	36.0 (35.0-38.0)	33.0 (29.5-34.0)	0.380	<0.001	30.0 (26.8-31.2)	30.0 (27.5-33.0)	33.0 (30.5-37.0)	35.0 (33.8-37.0)	38.5 (31.2-39.0)	0.459	<0.001
**LVEDD**	47.0 (44.0-51.0)	43.0 (41.0-46.0)	46.5 (43.0-49.8)	48.0 (45.0-51.0)	50.5 (47.8-51.8)	51.0 (48.5-53.0)	0.373	<0.001	44.5 (39.8-46.2)	45.0 (42.0-49.2)	48.0 (45.8-50.0)	50.0 (48.0-52.0)	52.0 (49.0-55.0)	0.521	<0.001
**TAPSE**	24.0 (21.0-27.0)	24.0 (23.0-27.0)	24.5 (22.2-26.0)	23.0 (21.0-26.0)	24.5 (19.2-29.0)	27.0 (21.0-27.0)	-0.065	0.530	23.5 (22.8-26.0)	23.0 (20.8-27.0)	24.0 (21.0-27.0)	24.0 (22.0-26.0)	26.0 (23.0-29.0)	0.086	0.406
**Ferritin**	60.0 (30.2-97.8)	42.5 (28.5-67.8)	69.0 (39.0-116.5)	58.5 (29.8-97.2)	76.5 (61.8-90.5)	72.0 (40.5-114.0)	0.136	0.183	72.0 (22.0-98.0)	43.0 (27.5-61.0)	65.0 (32.0-92.2)	70.0 (49.0-105.0)	83.0 (45.0-118.0)	0.266	0.008
**Transferrin**	2.8 (2.5-3.1)	3.0 (2.5-3.2)	2.7 (2.4-3.0)	2.8 (2.5-3.0)	2.6 (2.4-2.9)	3.0 (2.7-3.6)	-0.077	0.446	3.0 (2.6-3.4)	3.0 (2.7-3.3)	2.8 (2.5-3.0)	2.6 (2.4-2.8)	2.7 (2.4-2.9)	-0.390	<0.001
**Transferrin saturation**	26.0 (19.0-36.0)	23.0 (15.2-27.8)	20.0 (18.5-31.0)	27.5 (19.0-35.0)	35.0 (24.0-43.0)	36.0 (27.5-37.5)	0.220	0.034	18.0 (16.2-26.2)	27.0 (20.0-36.0)	30.0 (18.0-37.0)	24.5 (19.8-33.5)	34.0 (26.0-37.0)	0.244	0.018
**Soluble transferrin R**	3.0 (2.7-3.7)	3.2 (2.9-3.8)	3.0 (2.7-3.5)	3.2 (2.6-3.7)	2.6 (2.2-3.6)	2.8 (2.8-2.8)	-0.142	0.199	3.0 (2.9-3.7)	3.0 (2.7-3.8)	3.2 (2.8-3.8)	3.2 (2.5-3.3)	2.8 (2.5-3.2)	-0.136	0.218

n=100 athletes (one most recent examination per raw clinic-number identity among records with both scores). Continuous variables are median (IQR) unless Shapiro–Wilk normality held in the whole sample and in every Achievement and Endurance stratum and its complement, in which case mean ± SD is shown. Correlations are Spearman rank coefficients (exploratory).

Abbreviations: AS: Achievement Score, ES: Endurance Score, h: hours, HR: heart rate, VO2 max: maximum oxygen consumption, max: maximum, O2: oxygen, SMM: skeletal muscle mass, PBF: percentage of body fat, EF: ejection fraction, LA: left atrium, LVESD: left ventricle end-systolic diameter, LVEDD: left ventricle end-diastolic diameter, TAPSE: tricuspid annular plane systolic excursion, R: receptor.
